# Dysregulations of Functional RNA Modifications in Cancer, Cancer Stemness and Cancer Therapeutics

**DOI:** 10.7150/thno.41687

**Published:** 2020-02-10

**Authors:** Mohammad Burhan Uddin, Zhishan Wang, Chengfeng Yang

**Affiliations:** Department of Toxicology and Cancer Biology, College of Medicine, University of Kentucky, Lexington, KY 40536-0305, USA.

**Keywords:** epitranscriptomics, functional RNA modifications, mRNAs, non-coding RNAs, RNA modification machinery, carcinogenesis, cancer stem cells (CSCs)

## Abstract

More than a hundred chemical modifications in coding and non-coding RNAs have been identified so far. Many of the RNA modifications are dynamic and reversible, playing critical roles in gene regulation at the posttranscriptional level. The abundance and functions of RNA modifications are controlled mainly by the modification regulatory proteins: writers, erasers and readers. Modified RNA bases and their regulators form intricate networks which are associated with a vast array of diverse biological functions. RNA modifications are not only essential for maintaining the stability and structural integrity of the RNA molecules themselves, they are also associated with the functional outcomes and phenotypic attributes of cells. In addition to their normal biological roles, many of the RNA modifications also play important roles in various diseases particularly in cancer as evidenced that the modified RNA transcripts and their regulatory proteins are aberrantly expressed in many cancer types. This review will first summarize the most commonly reported RNA modifications and their regulations, followed by discussing recent studies on the roles of RNA modifications in cancer, cancer stemness as wells as functional RNA modification machinery as potential cancer therapeutic targets. It is concluded that, while advanced technologies have uncovered the contributions of many of RNA modifications in cancer, the underlying mechanisms are still poorly understood. Moreover, whether and how environmental pollutants, important cancer etiological factors, trigger abnormal RNA modifications and their roles in environmental carcinogenesis remain largely unknown. Further studies are needed to elucidate the mechanism of how RNA modifications promote cell malignant transformation and generation of cancer stem cells, which will lead to the development of new strategies for cancer prevention and treatment.

## Introduction

The canonical concept of RNA structure made merely of A, C, G and U nucleotides has proven overly simple with the discovery of modifications in these basic building blocks. In recent years extensive research in RNA biology has revealed diverse modifications of RNA post-transcriptionally. The history of studying RNA modification is as old as six decades. The first RNA modification was discovered in 1956 by a group of scientists in the University of California School of Medicine, Barkeley; where they isolated, purified and characterized the modified RNA in a NaCl soluble form 1, 2. The advent of whole genome and transcriptome sequencing as well as deep sequencing techniques has made the scenario more panoramic and versatile 3. It is now clear that the transcriptional outputs of the genes not only produce the messenger RNAs (mRNAs) but also produce non-coding RNAs that are not translated into proteins. The non-coding RNAs are briefly categorized into house-keeping non-coding RNAs (such as transfer RNAs), small non-coding RNAs (<200 nucleotides in length, such as microRNAs) and long non-coding RNAs (>200 nucleotides in length) 4. Although these non-coding RNAs do not participate in protein coding, they play a vital role in diverse biological processes. The diversity of their function stems from their expression patterns, stability, distribution and localization in the subcellular level, structural stability and pattern of splicing 5.

Precursor mRNA (pre-mRNA) undergoes a series of intricate processing of capping, splicing, polyadenylation to become mature mRNA and transport to cytoplasm in order for a gene to be translated into protein. This complex array of processes could be affected by a number of RNA binding proteins (RBPs). The binding specificity of RNA to RBPs is determined by the post-transcriptional modifications of the RNAs which are accomplished by addition or removal of certain chemical groups to the pertaining nucleotides 6. Modifications of these nucleotides not only change the identity of the molecule but also bring about changes in the biological role of the modified RNAs. These modifications occurring post-transcriptionally also affect the folding patterns of RNAs which are especially important for tRNAs and rRNAs to maintain their functional structures. For mRNAs and long non-coding RNAs (lncRNAs), the modifications determine their stability and binding specificity for the proteins while for the small regulatory RNAs such as siRNA and microRNAs (miRNAs) modifications are important to maintain their regulatory role by limiting the accessibility to their binding sites 7.

Recent advances in the RNA mapping technology has revealed the detailed map of several RNA modifications with the help of specific antibody coupled immunoprecipitation technique or chemical modifications followed by next generation sequencing 8-10. These techniques highlighted the dynamicity of RNA modification in controlling cellular processes ranging from gene transcription to translation into proteins. The dynamicity of RNA modification facilitates the cells to respond rapidly for adaptation to the changes in their microenvironment. This is especially important for the cells that undergo malignant transformation in cancer. Similar to the normal cells, tumor cells also adopt changes for survival and to avoid stress imposed by the components of the internal microenvironment or external stressors 11. In this review we will first summarize the most commonly-reported RNA modifications and their regulations; then we will discuss about the contributions of RNA modifications and their regulatory machinery in cancer. We will also highlight on how changes in RNA modification landscape are critically involved in generation of cancer stem cells (CSCs) and the potential of RNA modification machinery as cancer therapeutic targets. Future studies on the role of functional RNA modifications in cancer are proposed.

## RNA Post-transcriptional Modifications

**Figure 1:**
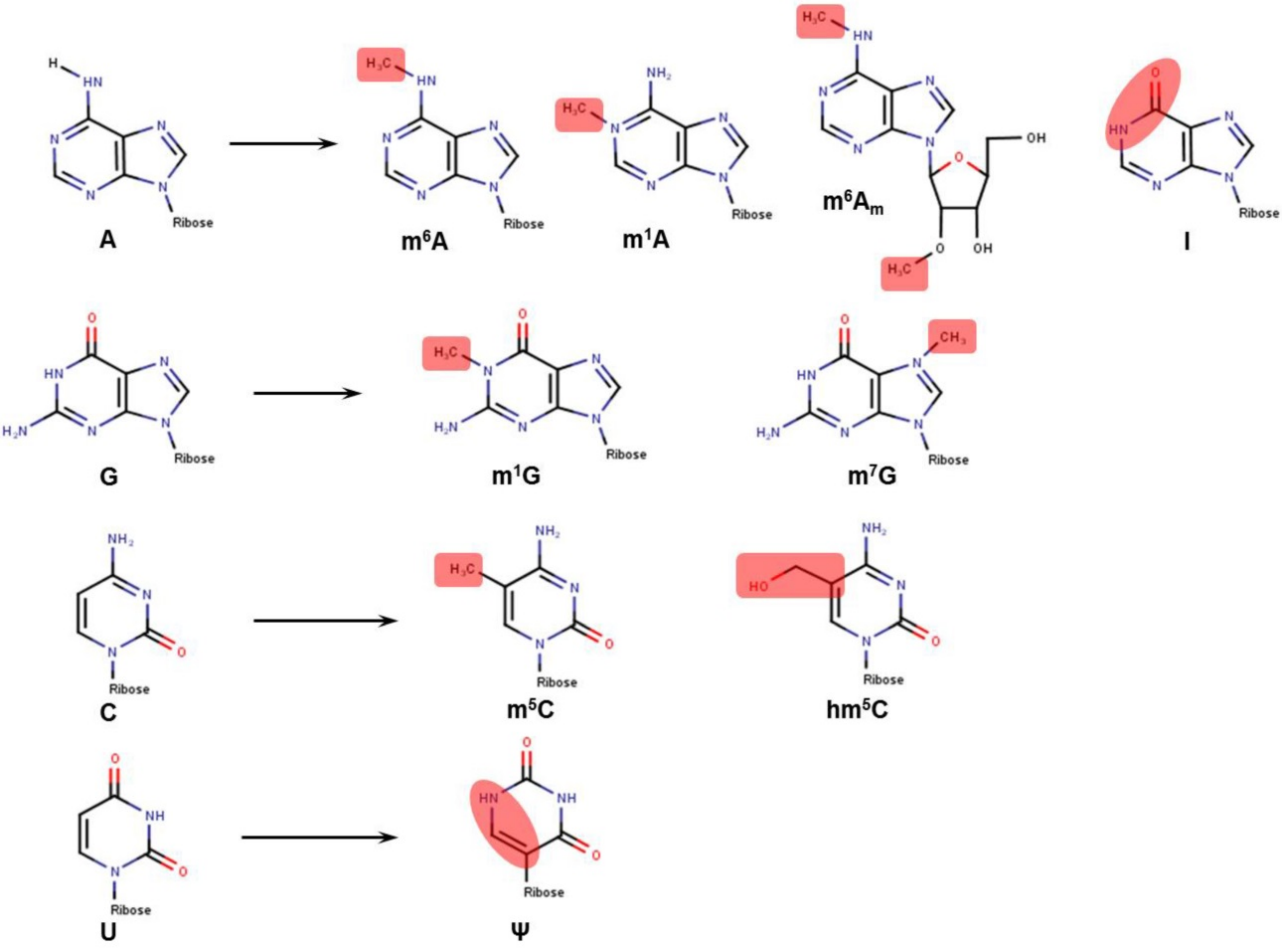
** Common modifications in four nucleotide bases present in RNA.** Chemical structures of some selected modifications are represented. The modification sites on the chemical structures are highlighted in red.

As a key player in the cellular processes, RNA plays critical roles in carrying the genetic information, catalyzes various biochemical reactions, carries vital information for protein synthesis by acting as an adapter and making the necessary scaffold for attaching the amino acids in proper sequential orders. The eukaryotic RNA contains different kinds of modifications as documented by several literatures. RNA modifications occur in all four nucleotide building blocks of RNA: A, C, G and U (Figure 1). By far approximately 163 modifications have been identified and more and more are being discovered. RNA modifications have been observed in all types of RNAs but are found most abundantly in noncoding RNAs (ncRNAs) such as ribosomal RNAs (rRNAs) and transfer RNAs (tRNAs). However, modifications are also reported in other types of RNAs including mRNAs, lncRNAs, small nuclear RNAs (snRNAs) etc., although to a much lower abundance. These modifications are important for maintaining vital roles of RNAs in translation and splicing 12. At the beginning the RNA modifications were thought to be irreversible, but recent discoveries have shown that at least some of the modifications are reversible 13. The discovery of reversible RNA modifications has coined the terms 'RNA epigenetics' and 'epitranscriptomics' in RNA research that are being extensively used now a day and has become the talk of the table 9, 14. Although more than hundred modifications are identified so far, only few of them are well studied. For most of them their normal cellular functions as wells as their involvements in pathological conditions are unknown. For our review we only highlighted on few of the well-studied RNA modifications which have been reported to be involved in vital cellular physiological processes as well as implicated in cancer.

### N6-methyladenosine (m^6^A)

The m^6^A RNA methylation is the most abundant mRNA modification in eukaryotic cells (about 0.1 to 0.4% of the total number of adenosine), which plays crucial roles in RNA fate and metabolism 15. It was first discovered in 1974 which is found in all arrays of life- from eukaryotic cells down to prokaryotes 16. The m^6^A is present in high abundance in mRNA although it can also be found in noncoding RNAs such as tRNAs, rRNAs 17, lncRNAs 18 and snRNAs as well 19. In higher eukaryotes on an average each mRNA molecules contain 3-5 m^6^A sites 14. Before the discovery of the m^6^A methylation in mRNA, the whole field of RNA research was focused mainly on tRNAs, rRNAs, and snRNAs which are highly expressed and catalytically active types of RNAs. The underlying reason for this lacking is the technical difficulties in mapping the m^6^A modifications in mRNA transcripts, the expression level of which is very low (only ~3% of the total RNA) 20, 21.

**Figure 2:**
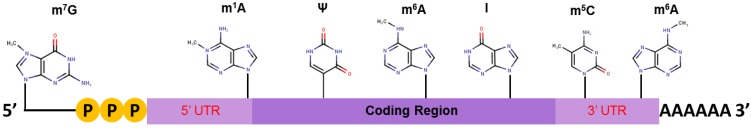
** Locations of different modifications in mammalian mRNA.** 5'UTR: 5' untranslated region; 3'UTR: 3' untranslated region.

One of the most important features of the m^6^A modification is that, the m^6^A modification is confined to the consensus motif RRACH; where, R represents G or A, A is m^6^A and H represents A, C or U. This consensus sequence may occur every 85 nucleotides, thus on an average each mRNA may contain approximately 30 of such sequence sites. Since only 3-5 m^6^A sites are assumed to be present per mRNA, adenosine in most of the consensus sequences probably are not modified or may be modified in special circumstances. m^6^A modifications are not equally distributed throughout the entire transcriptome, instead they are preferentially enriched in the sites of consensus motifs which are located mostly near the stop codons at 3' UTRs and long internal exons. The m^6^A modifications are conserved throughout the entire human and mouse embryonic stem cell transcriptome 22, 23. The methylation of RNA molecules occurs most commonly in the base itself and the 2'-OH group. The chemical property of the modified nucleotide is similar to its normal counterpart which makes it almost impossible to differentiate through chemical processes 23. With the advent of transcriptome-wide mapping of m^6^A through immunoprecipitation of m^6^A enriched mRNA with m^6^A-specific antibody followed by sequencing has enabled us to identify the more abundant m^6^A regions on the gene. In 2012 two research groups independently described the methods to determine the genome-wide distribution of m^6^A methylated mRNA 9, 24. They exploited the novel approach of fragmenting the total cellular RNA to ~100 nucleotide fragments and immunoprecipitating the m^6^A-rich fragments using m^6^A targeted antibody followed by a high-throughput sequencing (MeRIP-seq). The mRNA fragments thus isolated were then reversely transcribed and the resulting cDNAs were amplified by PCR and sequenced to determine the extent of mRNA containing the methylation. This method which detects the m^6^A in a ~100-nucleotide resolution, reveals that m^6^A RNA modification is distributed mainly in the coding and the 3' untranslated region (3'-UTR) (Figure 2). This method has shown that, m^6^A is ubiquitously distributed among the entire transcriptome occurring approximately 7,000 sites in mRNA and about 300 sites in non-coding RNAs (ncRNAs). Based on the mutational studies and methyltransferase substrate preferences, the m^6^A contents in the mammalian cells were found to be an estimate of 0.1-0.4% of the total adenine present 15.

The antibody-based identification method of m^6^A has been widely used to quantify the overall transcriptome wide distribution of m^6^A. To determine the presence of m^6^A in the individual nucleotide resolution level, newer methods have been developed with higher resolution. One such method is called SCARLET (site-specific cleavage and radioactive-labeling followed by ligation-assisted extraction and thin-layer chromatography) which can site-specifically quantify the m^6^A in individual nucleotide resolution level 18. Modifications of the antibody-based method is also used to detect m^6^A in the individual-resolution level. Using the antibody-specific recognition technique Linder et al. developed a method called 'm6A individual-nucleotide-resolution cross-linking and immunoprecipitation (miCLIP)'. The basis of specificity of this method is UV-induced crosslinking of the antibody and RNA which upon reverse transcription results in mutated or truncated cDNA at the site of crosslinking. Thus the position of the modification can be detected from the presence of antibody-bound nucleotide 25. However, the antibody-based methods lack specificity and other potential drawbacks which prompted the development of non-antibody based methods. Other methods developed to quantify m^6^A in individual level include the use of m^6^A-sensitive reverse transcriptase 26, substitution of the allyl for methyl group with methyltransferase enzymes followed by mutagenesis 27 and use of deoxythymidine triphosphate (dTTP) analog 4SedTTP 28. Recently Zhang et al. has introduced a more advanced single-base resolution method with more accurate measurement of m^6^A. Using the m^6^A sensitive RNA endoribonuclease, MazF and ChpBK, they developed a new method called m^6^A-sensitive RNA-endoribonuclease-facilitated sequencing or m^6^AREF-seq. This newly developed method facilitated to quantify the methylation level with single-base resolution in order to identify the m^6^A sites at specific motifs 29. Another antibody-free m^6^A detection method is the 'deamination adjacent to RNA modification targets followed by sequencing (DART-seq)'. This method takes advantage of the cytidine deaminase APOBEC1, which is in the CRISPR-Cas9 genome editing technique that causes alterations of cytosine to uracil (C to U) at the target sites in the single-stranded DNA. In this technique APOBEC1 is fused with the m^6^A-binding YTH domain of YTHDF2 protein which is then subjected to the RNA sample. APOBEC1 deaminates the cytosine present immediately following the m^6^A residue in the consensus sequence which is detected by reverse transcription followed by high-throughput sequencing 30. In summary, these transcriptome-wide m^6^A mapping techniques have shown that these m^6^A modified regions are versatile in all over the transcriptome with widespread biological roles in the living systems.

### 5-methylcytosine (m^5^C)

The 5-cytosine methylation is a widespread DNA modification in eukaryotes but is also reported to be present in different types of RNAs, mostly in noncoding RNAs- tRNAs and rRNAs. It was first discovered in DNA in early 1950s and was later identified in RNA. m^5^C was first detected in mRNA almost at the same time with m^6^A in 1970s although in much less abundance 3. In genomic DNA, specific locations of m^5^C could be quantitatively determined with bisulfite sequencing technology. Taking advantage of this technique, transcriptome-wide m^5^C modification map could also be determined in RNAs. m^5^C modification was found more abundantly in tRNA among all other RNAs where the modification most commonly occurs in the variable region and the anticodon loop. While in rRNA, m^5^C could be found in different ribosomal subunits which is catalyzed by the SAM-dependent methyltransferase enzymes 31. In addition to tRNA and rRNA, m^5^C modification has also been identified with mammalian mRNA and non-coding RNAs. Over ten thousands m^5^C modification sites have been identified in RNA isolated from HeLa cells with bisulfite conversion followed by high throughput sequencing. The principle behind this method is the chemical conversion of unmodified cytosine residues with the treatment of sodium bisulfite to uracil and identifying the intact cytosine as m^5^C candidate. The modified cytosine is identified as C by reverse transcription while the unmodified cytosine is identified by presence of T in the resulting cDNA 32. However, bisulfite treatment has several serious drawbacks. First, it cannot convert the cytosine in the double stranded regions of tRNA. Second, it cannot distinguish the m^5^C from other cytosine modifications that are resistant to bisulfite treatment and is prone to include false positive results. Third, bisulfite treatment causes significant RNA degradation and therefore unable to pre-enrich m^5^C RNA fragments for sequencing 12. Modification of bisulfite sequencing method has been applied in a different study to identify the m^5^C modification in both eukaryotic, prokaryotic and archaeal RNA. This method was validated by direct immunoprecipitation of m^5^C RNA fragments followed by cDNA library preparation and massive parallel sequencing. This method not only identified novel m^5^C sites within the rRNA and tRNA along with the existing sites but also identified a consensus motif AUC(m^5^C)GANGU for m^5^C modification in archaeal mRNA 33.

**Figure 3:**
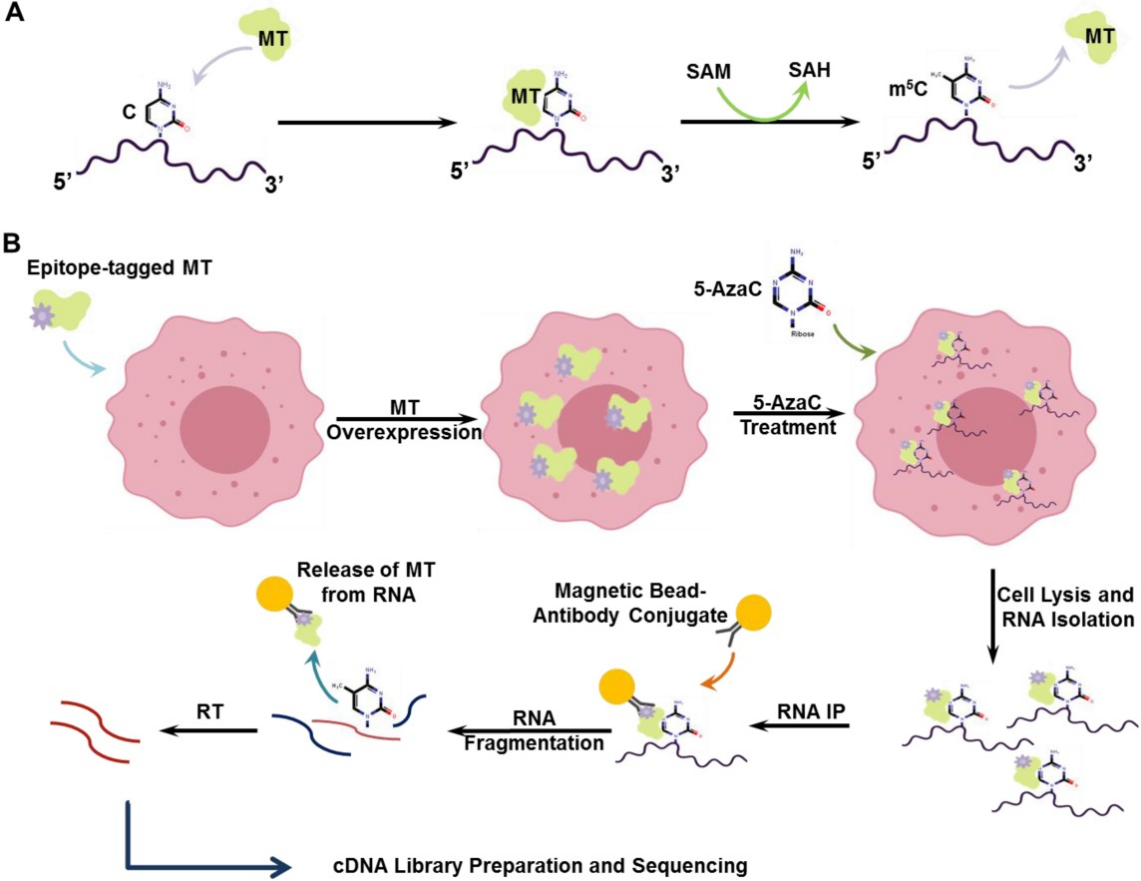
** A Schematic description of 5-azacytidine immunoprecipitation (Aza-IP) of m^5^C detection.** A. Mechanism of cytosine methylation: the methyltransferase (MT) catalyzes the transfer of methyl group to cytosine from SAM. B. Mechanism of Aza-IP -based m^5^C mapping: epitope-tagged MT is overexpressed inside the cell which is treated with 5-AzaC. 5-AzaC is incorporated in the nascent RNA which irreversively binds to MT. Epitope-tagged MT is captured and immunoprecipitated along with the bound RNA which is then released and the RNA is fragmented. The RNA is reversely transcribed and cDNA library is prepared followed by sequencing.

To accurately determine the m^5^C positions in RNA, a mechanism-based approach named 5-azacytidine mediated RNA immunoprecipitation (Aza-IP) has been developed. This method exploits the catalytic activity of the m^5^C methyltransferases to link with its RNA substrate and subsequent methyl transfer from S-Adenosyl Methionine (SAM) (Figure 3a). This methyl transfer is disrupted by 5-azacytidine which forms covalent bond with the methyltransferase. 5-azacytidine is randomly incorporated in the nascent RNA in overexpressed epitope-tagged m^5^C methyltransferase cells which is then immunoprecipitated by an antibody specific for the epitope-tagged m^5^C methyltransferase. After the fragmentation and purification of immnuprecipitated RNA, cDNA library is prepared which is then sequenced and mapped for m^5^C sites (Figure 3b) 10. In another study, the modified individual-nucleotide-resolution crosslinking and immunoprecipitation (iCLIP) named methylation iCLIP (miCLIP) was used to identify the transcriptome-wide m^5^C sites. In substitution of UV crosslinking, a site-specific mutation is carried out on the target RNA which stabilizes the methyltransferase-m^5^C RNA complex and is immunoprecipitated that can be detected by western blotting. The RNA from this complex is then isolated and amplified for library preparation which is followed by sequencing. With this technique m^5^C sites could be identified in known positions and in positions previously unknown in tRNA and rRNAs as well as mRNAs and other ncRNAs 34. Based on these findings from sequencing, the m^5^C modified bases are more abundantly found near the UTRs (Figure 2), particularly near the Argonaute proteins I-IV binding sites compared to other locations 32. Although most of our knowledge about m^5^C modification comes from the study of tRNA and rRNA, advances in sequencing techniques have revealed the presence of m^5^C in other forms of RNAs such as- mRNA, snRNA (small nuclear RNA), miRNA, lncRNA etc.

### N1-Methyladenosine (m^1^A)

The m^1^A, another frequently occurring RNA modification was first discovered in 1961 and was isolated from the RNA sample in the following year by the same research group 35. Methylation on the N1 position of adenosine produces the modified nucleotide m^1^A which diminishes the Watson-Crick base pairing of A to T or U because of the steric hindrance by the methyl group. This hindrance of base pairing in the m^1^A-T/U interface may result in a misincorporation of the base by unstable Hoogsteen base pairing with other nucleotides or truncation of the nucleotide chain. N1-methylation of A produces a positive charge on the m^1^A molecule which can dramatically alter the RNA structure or alter the RNA-protein interaction 3, 36. By far the presence of m^1^A has been identified in rRNA, tRNA and most recently in mRNA 8, 37. m^1^A is versatile in almost all organisms from prokaryotes like bacteria, archaea to humans. This modification is most commonly found in the tRNAs at positions 9 in metazoan mitochondrial tRNAs, position 14, and 58 in metazoan tRNA T-loop 37, 38. In human m^1^A was identified in position 9 and 58 of the tRNAs in cytoplasm and mitochondria whereas at position 1322 on 28S subunit of rRNA 39, 40.

Research on m^1^A RNA modification in mammalian mRNA so far has seen very slow progress. The main reason behind this is, the abundance of mRNA is much less compared to the other types of RNA and the m^1^A modification in mRNA is even lower than that of tRNA (the m^1^A to A ratio is ~ 0.015%-0.054% in mammalian cells) 35. Also there is lack of methods that could adequately detect and quantify the extent of m^1^A methylation in the transcriptome with adequate sensitivity and reproducibility. Similar to the detection of m^6^A in mammalian mRNA, Dominissini et al. adopted an m^1^A-specific antibody binding approach where they separated the antibody bound m^1^A-enriched mRNA fragments and carried out reverse transcription. They characterized the m^1^A-carrying mRNA by the nature of the modification to introduce mismatched nucleotide and truncation and identified the mismatch rate created by m^1^A using Dimroth rearrangement (m^1^A converted to m^6^A) 8. A similar approach using the m^1^A-specific antibody for detecting m^1^A in mRNA has been adopted by Li et al., but they used the demethylase enzyme ALKBH3 to convert m^1^A to A instead of Dimroth rearrangement. Exploiting the tendency of m^1^A to produce truncated RT products, they were able to calculate the truncation positions 37. Another similar technique called 'm^1^A -ID-Seq' was introduced which also relies on m^1^A immunoprecipitation and the ability of the m^1^A to stall the RT reaction 41. With the help of these techniques, m^1^A was found to be highly enriched in the 5'UTR regions and close to the start codons (Figure 2). To identify the positions of m^1^A more precisely in a single-nucleotide resolution level a newer technique has been introduced which utilized a reverse transcriptase called TGIRT (Thermostable Group II Intron Reverse Transcriptase). This method made use of m^1^A -induced misincorporation in cDNA to identify mutational signature combined with pre-enrichment of m^1^A and *in vitro* demethylation. These all together ensured mapping m^1^A in human transcriptome with a higher level of sensitivity and confidence 42. Another protocol for single-nucleotide resolution m^1^A detection follows the basic m^1^A containing mRNA enrichment step with m^1^A immunoprecipitation and Dimroth rearrangement followed by TGIRT mediated reverse transcription 43.

### A-to-I (Adenosine to Inosine)

The Adenosine to Inosine (A-to-I) modification was first discovered in developing Xenopus embryo, the South African clawed toad (*Xenopus laevis*) 44. It is the enzymatic deamination of adenosine where the amine group is removed from adenosine base in a double stranded RNA (dsRNA). A-to-I RNA editing is most frequently observed in the repetitive sequences in mammalian transcriptome, i.e. Alu repeat elements which belong to the retroelement class called short interspersed nuclear element (SINE) in non-human mammals and in humans 45. In the living organisms, the deamination from adenosine occurs in two different mechanisms: a. Deamination through hydrolysis and b. Insertion of the inosine base hypoxanthine in adenosine. Initially, two enzymes- adenosine deaminase and AMP deaminase were identified as the catalyst for purine metabolism in biological system that are involved in replacing amino group with an oxygen atom from the water molecule. On the other hand, replacement of adenosine with hypoxanthine is carried out by the enzymatic cleavage of the glycosyl bond followed by removal of adenosine and insertion of hypoxanthine 46.

The adenosine usually base-pairs with uridine, but modification to inosine confers it structural similarity to guanosine (G). The inosine produced by the A-to-I modification is misread as G and thus base pairs with cytosine (C). The enzymes that recognize G, also read I as G and the protein thus translated changes the sequence read of the modified RNA. This mismatched base pairing also brings about structural conformational changes of the edited RNA, creating additional bulge or removing the existing ones from the dsRNA. This is because the modification can increase or decrease the base pairing through misreading of I as G 47. A-to-I RNA editing can occur in both coding and noncoding regions of any double stranded RNA (small regulatory RNA, mRNA etc.). Since A-to-I modification is widespread in living organisms ranging from metazoans to humans and plays vital biological roles, it is important to map the locations of the editing sites to better understand the implications of this editing. Bioinformatic studies have predicted several locations of A-to-I modification in all over the human transcriptome including intronic, exonic or even untranslated regions 45. Alu repeat elements in the untranslated regions and introns of human genome is the major target sites for the A-to-I modifying enzymes (ADARs) 48, 49. As I is read as G during the reverse transcription instead of A, the conventional method for detecting A-to-I modification site involved identification of A-to-G mismatch sites on the cDNA after the reverse transcription and comparing it with the genomic DNA sequence. This is a simple strategy which has been used for identifying the modification sites 50. But this strategy suffers from misinterpretation with background noises produced from the presence of somatic mutations, single nucleotide polymorphisms (SNPs), G contamination from pseudogene amplification or the errors in sequencing or mapping 51. Another approach for A-to-I identification was to knockdown or knockout of ADARs by RNAi. This approach measures the decrease in G peak ratios in cDNA sequence chromatogram after A to G replacement 52. However, knockdown or knockout of ADARs caused alteration in gene expression, phenotypic changes or even lethality in experimental animals rendering this method impracticable 53, 54. To avoid the limitations of these approaches and accurately mapping A-to-I modification in human transcriptome, Sakurai et al. developed a biochemical method which they named as inosine chemical erasing (ICE). In this method they carried out cyanoethylation of I with acrylonitrile to produce N^1^-cyanoethylinosine (ce^1^I) which prevents I to base pair with C. This avoids false positive G peaks in the cDNA chromatogram 55. Although this method proved to be a robust method to identify A-to-I location, it still has some limitations, i.e. it is limited to detection of only some site specific regions instead of transcriptome-wide identification. To overcome this limitation they took advantage of advanced RNA sequencing technology and developed the modified ICE-seq method. With the help of this method they were able to identify ~20,000 novel A-to-I modification sites in human transcriptome 56.

### Pseudouridine/ 5-ribosyluracil (Ψ)

Pseudouridine (Ψ) is the first discovered nucleoside present in RNA and is the most abundant RNA modification, the ratio of Ψ/U being 7-9% in the entire transcriptome. Structurally it is the 5-ribosyl isomer of the uridine base of RNA which is often termed as “the fifth nucleoside” 57. Ψ modification is unique among all other RNA modifications because of the nature of bond between the base and the sugar moiety created inside the molecule. The glycosidic bond between the base and ribose sugar in uridine undergoes breakdown and reformation followed by 180^0^ rotation of the base gives rise to its isomeric form, Ψ. Thus, in Ψ structure a C-C bond appears in place of typical N-C bond between the ribose sugar and the base as seen with other nucleosides. This conformational change creates an extra H-bond donor on N1 position of the Ψ molecule. This additional N1-H forms H-bond with a water molecule and contributes to base stacking due to more bonding interaction with other molecules. This unique feature provides the molecule with enhanced conformational rigidity and thermodynamic stability 58, 59. Although these differences between the uridine and Ψ do not affect the base-pairing specificity; they significantly affect the base-pairing stability, base stacking and the tertiary structure of the RNA molecule carrying this modification 60.

Ψ is most abundantly present in noncoding RNAs such as rRNAs, tRNAs, snRNAs and small nucleonar RNAs (snoRNAs). However, recent studies revealed the presence of Ψ in lncRNA and mRNA transcripts as well 59. With quantitative MS analysis Li et al. revealed a wide-spread abundance of Ψ in mammalian mRNA (the Ψ/U ratio being approximately 0.2-0.6%) 61. In tRNA, Ψ is universally present in the 55 position of the TΨC stem-loop structure. It is also present in other locations such as at postion 13 on the D stem, at postion 39 on the anticodon stem and at postion 38 on the anticodon loop structure. Presence of Ψ in these locations confers the tRNA structural stabilization, codon-anticodon recognition and ensures overall translational fidelity 62. In rRNA, Ψ is ubiquitously present in all domains of life from archaea to eukaryotes. Ψ is present in different functionally important regions on rRNAs such as peptidyltransferase center, decoding center and the sites for interaction with mRNA and stem-loop structure of tRNA in both small and large ribosomal subunits. Presence of Ψ on rRNA provides additional H-bonding which contributes to proper folding of rRNA structure through base stacking. Ψs thus play vital roles in protein synthesis by regulating peptidyl transfer, codon-anticodon recognition and decoding amino acid information 57, 62. In snRNA, the Ψ modified residues are present in the locations which are important for RNA-RNA and RNA-protein interactions. Presence of Ψ on snRNA controls the assembly of splicing factors and proper positioning of spliceosomes for their optimal function. Base pairing between Ψ on snRNA and A on the pre-mRNA intronic junction stabilizes the snRNA/pre-mRNA complex to facilitate mRNA splicing 57.

Since Ψ is the most abundant RNA modificantion which has been identified in almost all forms of RNAs, the functional consequences of this modification could be better understood with accurate and reliable transcriptome-wide mapping. Until now the most widely used method for detection of Ψ modification relies on its ability to form a stable complex with 1-cyclohexyl-(2-morpholinoethyl) carbodiimide metho-p-toluene sulfonate (CMCT). CMCT reacts with specific N atoms on the G and U where it forms carbodiimide (CMC) adduct with the N1 on G, N3 on U and both N1 and N3 on Ψ. After the alkaline treatment at a pH ~10.4, all the CMC adduct is hydrolyzed except N3 on Ψ. The bulky CMC group attached to N3 on Ψ hinders the reverse transcription and thus results in a cDNA truncation. This facilitates the detection of Ψ in a single nucleotide resolution level 63. Based on the principle of reverse transcription stop, a high-throughput method called pseudouridine site identification sequencing (PSI-seq) was developed to identify the presence of Ψ in yeast mRNA transcriptome. In this method the truncated cDNAs produced due to reverse transcription stop by CMC adduct was used for library preparation followed by deep sequencing 60. Modification of CMC based method is also applied to qualitatively and quantitatively determine the presence of Ψ. This modified CMC-Ψ based method utilizes a new RT condition that can read both the mutation and deletion on the cDNA products. The RT reaction results in different melting curves based on mutation/deletion which facilitates the detection of Ψ 64. Recently, an RTqPCR based method called CMC-RT and ligation assisted PCR (CLAP) was developed to quantitatively detect the Ψ sites in the mRNA and lncRNA. This method also uses CMC-Ψ induced RT stop with an additional step of site-specific ligation followed by PCR to generate two unique PCR products that correspond to the modified and unmodified uridine. The modification is thus visualized in PCR products through gel electrophoresis 65. In addition to CMCT based detection, RNA bisulfite sequencing can also detect the Ψ site in a single nucleotide resolution level. A modified bisulfite sequencing named RBS-Seq has been introduced to detect Ψ simultaneously with other RNA modifications like m^5^C and m^1^A 66.

## Other modifications in RNAs

Besides N6‑methyladenosine (m^6^A), 5‑methylcytosine (m^5^C), N1‑methyladenosine (m^1^A), Adenosine to Inosine (A to I) and pseudouridine (Ψ) modifications that are sparse and versatile in the entire transcriptome, there are hundreds of other RNA modifications and more are likely to be identified. The modifications exhibit either simple addition of a methyl or acetyl group or rearrangement of the molecule giving rise to a modified isomer. However, more complex modifications involving multiple chemical modifications in the same molecule also exist.

### N6,2´-O-dimethyladenosine (m^6^A_m_)

Shortly after the discovery of m^6^A, a serendipitous dimethylated adenosine modification was discovered by Wei and colleagues. While studying the other 2′-O-methylated cap nucleotides, they observed the base-methylated version of 2′-O-methyladenosine (A_m_) which they named m^6^A_m_. The additional methylation occurred at the N6 position of A_m_ 67. Typically, the 5' end of the mRNAs in eukaryotes consist of m^7^G cap. The nucleotides next to the m^7^G of the transcribed mRNA are usually methylated on the ribose sugar at the 2'-OH position. The A_m_ present at that position may undergo an additional methylation at N6 to form m^6^A_m_. m^6^A_m_ is thus located mainly at the beginning of the transcribed nucleotide positioned in mRNA which is in vicinity of m^7^G. While m^6^A is identified as the most common internal modification of mRNA, m^6^A_m_ is discovered as the second nucleotide from beginning of the 5' UTR of mRNA (m^7^GpppN_m_). The abundance of m^6^A_m_ is very low in the 5' UTR of mRNA, the ratio of m^6^A_m_ to m^6^A ranges between 1:10 to 1:15 68.

Being very similar in chemical structure with m^6^A, it is very difficult to chemically identify m^6^A_m_. MeRIP-Seq approach for transcriptome wide mapping of m^6^A does not work for identification of m^6^A_m_ since this approach uses m^6^A-specific antibody which binds with both m^6^A and m^6^A_m_. To identify m^6^A_m_ with high resolution, a miCLIP method has been introduced which is the modified version of individual-nucleotide resolution cross-linking and immunoprecipitation (iCLIP) approach. Similar to iCLIP, this method uses UV crosslinking of anti-m^6^A antibodies to RNA and a miCLIP library is prepared which is then used for subsequent steps to map m^6^A and m^6^A_m_ residues in a single nucleotide resolution level. Without miCLIP the peak signal within the 5′ UTR would refer to m^6^A instead of m^6^A_m_ which is found only in the transcription start site. In miCLIP technique the m^6^A_m_ present in 5′ end of the RNA is protected through library preparation which is not ensured in other techniques. By this way the ambiguity for misannotation of m^6^A for m^6^A_m_ is eliminated 69.

### m^7^G

m^7^G is present in the cap structure of almost all eukaryotic mRNAs which is installed during the transcription initiation and is involved in regulating mRNA stabilization, transport and splicing. This is also one of the internal modifications of non-coding RNAs such as tRNAs, rRNAs, miRNAs etc. occurring most commonly in position 46 of the tRNAs in both yeast and human and position 1639 of the 18S subunit of the human rRNA 70-72. Like other common internal modifications (e.g. m^6^A, m^5^C) m^7^G-MeRIP technique has been used taking advantage of the m^7^G-specific antibody for transcriptome-wide mapping of m^7^G 73. For more precise identification of m^7^G with higher resolution, a chemical modification based approach called tRNA reduction and cleavage sequencing (TRAC-Seq) has been developed recently 74.

With the advent of novel detection techniques, the overall transcriptomic mapping of these modifications is slowly being revealed. One of the techniques that brought about unprecedented insight in posttranscriptional RNA modification and revolutionized our understanding in this field is the antibody-based detection method. This method, although emerged as an efficient technique in recent years for detecting m^6^A and m^5^C in the RNA transcriptome globally, however; is associated with major drawbacks and is not immune to error. One of the major disadvantage of this technique is the requirement of large amount of RNA input. Another drawback for this method is the non-specific binding to the modified nucleotides that possess structural similarity, such as misrecognition of m^6^A for m^6^A_m_ by m^6^A specific antibody. The overall process is costly and time consuming, involving a lot of efforts for library preparation prior to sequencing 30. In order to comprehend the functional significance of these modifications, their abundance, location and dynamicity of regulation needs to be precisely determined. Although advancement of chemical and molecular biological methods have made significant contributions to this end, yet methods with high sensitivity for accurate quantification and single-base resolution with adequate precision remains a crucial determinant for the success in this area.

## Regulators of RNA Modifications: Writers, Erasers and Readers

RNA modification is a dynamic process, which at the beginning was thought to be irreversible. With the discovery of the RNA modifying proteins (writers, erasers and readers) it has become evident that, this process is reversible and well regulated. Although there are more than hundred different RNA modifications that has been discovered until now, the regulatory proteins for all of the modifications have not been identified yet. For some of the modifications, only the proteins that are installing the modifications have been identified (Table 1). In this review, we limited our discussion only with the modification regulators that are related to our context.

### Writers

#### m^6^A

Discovery of the involvement of m^6^A modifications in gene transcription was a major breakthrough in molecular biology. The posttranscriptional installment of m^6^A is accomplished by a multicomponent methyltransferase complex which are commonly termed as m^6^A 'writers'. In mammalian cells this complex contains METTL3 (also known as MT-A70), METTL14, and WTAP 23, 75. METTL3 was first identified in 1997 by enzymatic digestion of a 200 kDa methyltransferase complex from the HeLa cell nuclear extract. This methyltransferase is a homologue of the inducer protein of meiosis in yeast, IME4 which is a subunit of 1MDa protein complex 22. The second methyltransferase METTL14 was later identified as another writer molecule in the methylation complex. Structural analysis of METTL14 methyltransferase domain was found to be phylogenetically related to the METTL3 and considered as homolog of METTL3.

**Figure 4:**
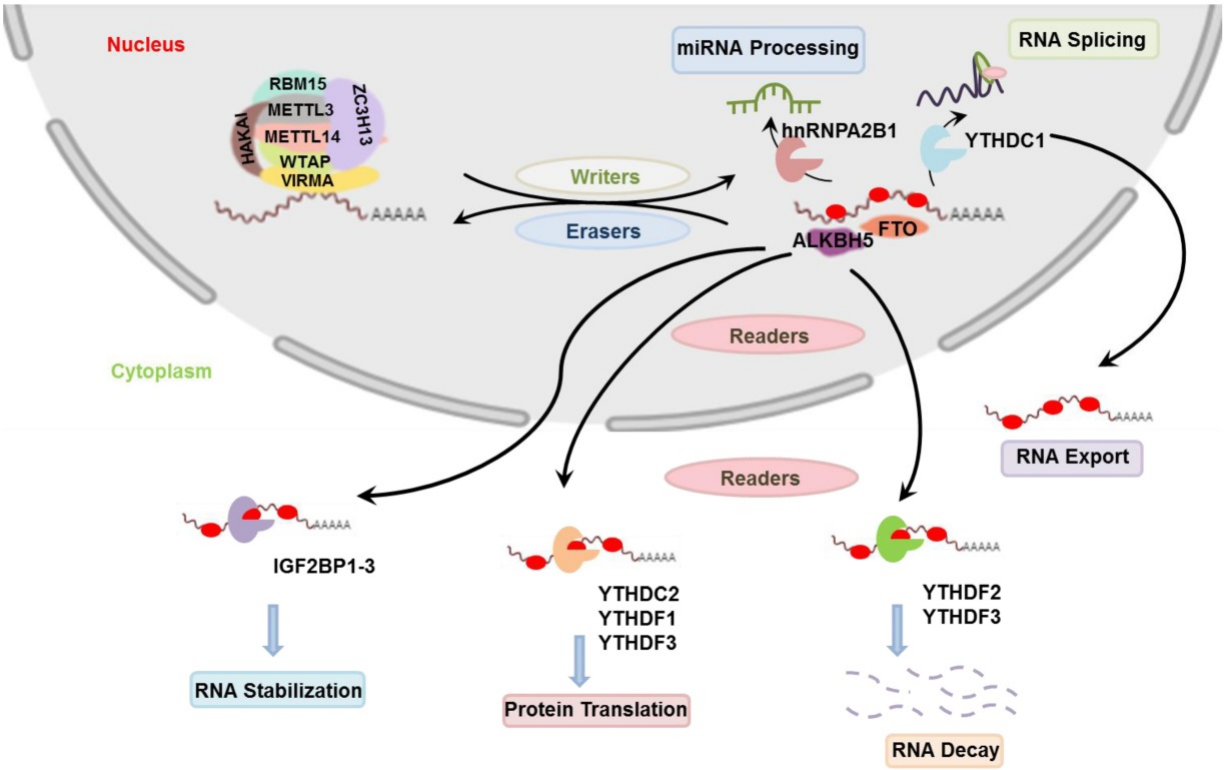
** Regulators of m^6^A RNA modification: writers, erasers and readers.** The reversible m^6^A RNA modification is carried out by the writer proteins that act on the target RNA transcript in the form of multimeric complex containing METTL3, METTL14, WTAP, VIRMA, HAKAI, ZC3H13 and RBM15. The modification is reversed by the erasers (FTO and ALKBH5). The functional outcome of the modification is determined by the reader protein (includes- YTH family, hnRNPs and IGF2BP family proteins) that recognizes the modification. METTL3, Methyltransferase-like 3; METTL14, Methyltransferase-like 14; WTAP, Wilms tumour 1‑associated protein; VIRMA, Vir-like m^6^A methyltransferase associated protein; ZC3H13, Zinc finger CCCH domain-containing protein 13; RBM15, RNA-binding motif protein 15; FTO, Fat mass and obesity-associated protein; ALKBH5, AlkB homologue5; YTH, YT521B homology; hnRNPs, Heterogenous ribonucleoproteins; IGF2BP, Insulin-like growth factor 2 mRNA-binding protein.

Knockdown of METTL14 resulted in a decreased level of m^6^A methylation which further by proteomic profiling and co-immunoprecipitation was found to be associated with METTL3. Both of the enzymes are capable of catalyzing the transfer of a methyl group from a co-factor called S-adenosyl methionine (SAM) to the GGACU and GGAUU sequences located in a single stranded or a stem-loop form of RNA 75, 76. The methyltransferase activity of individual METTL3 or METTL14 is almost undetectable, however; a synergistic methylation effect was observed in their complex form. Elucidation of the crystal structure of methyltransferase domain of the METT3-METTL14 complex revealed that, METTL3 is the main component of the complex playing the catalytic function; whereas METTL14 acts as a pseudo-methyltransferase by binding to RNA and stabilizing the METTL3-RNA interaction 23, 76, 77. The third component of the methylation writer complex, WTAP was identified as another essential protein for m^6^A installation which binds to Wilms' tumor protein 1, hence the name WTAP. Initially it was considered as a splicing factor regulating RNA splicing to control cell cycle progression and embryonic development. The involvement of WTAP in RNA methylation was discovered by gel filtration experiment where it was found to form a large molecular weight complex with METTL3-METTL14. With photoactivable ribonucleoside-enhanced crosslinking (PARCLIP) assay it was found that METTL3, METTL14 and WTAP share the same RNA binding motif by which all of them recognize the same m^6^A consensus sequence on RNA. Thus, in the mammalian cells, METTL3 and METTL14 complex perform the methylation on m^6^A whereas WTAP join the complex to affect the m^6^A deposition inside the cell 23, 75, 78. WTAP was also found to coordinate the nuclear localization of the METTL3-METTL14 complex thus facilitating m^6^A deposition, which established the notion that the m^6^A modification is dynamic and reversible in nature which further associated its link to human health 78. Relatively low stoichiometry of WTAP in the complex indicated its weaker interaction with them as compared to METTL3-METTL14 interaction. It doesn't have methylation potential when alone since it lacks the catalytic domain for methylation as found in the other two members. This heterotrimeric complex is colocalized in the nuclear speckles 75. Disruption of any one component of the complex leads to a reduction in the m^6^A level in mRNA. RNA-binding motif protein 15 (RBM15) and its paralogue RBM15B was also reported to act as a mediator of m^6^A methylation which recruits the methylation complex in specific modification sites in RNA 79. Recently few other methyltransferase components - Vir-like m^6^A methyltransferase associated (VIRMA, also known as KIAA1429), HAKAI and Zinc finger CCCH domain-containing protein 13 (ZC3H13) have been discovered. With RNA co-immunoprecipitation followed by mass spectrometry, these proteins were discovered as the new components of the complex interacting with METTL3-METTL14-WTAP core catalytic complex. In this large multi-component complex, VIRMA is assumed to serve as a scaffold to hold the WTAP, HAKAI, ZC3H13 together, which combinedly create a pocket for accommodating the METTL3 and METTL14 catalytic subunits (Figure 4) 80. While most of the m^6^A modification is carried out by the methyltransferase complex, an independent m^6^A writer is also reported, the METTL16. METTL16 was found to interact with various pre-mRNAs, lncRNAs and ncRNAs 81. Another paralogue of METTL3 and METTL14 methyltransferase, METTL4 was identified in some eukaryotes. It is primarily considered as a DNA methyltransferase, however in some organisms the absence of METTL3 and METTL14 speculates its role in RNA methylation. The role of METTL4 as an RNA methyltransferase is yet to be confirmed 82.

#### m^6^A_m_

The methytransferases that bring about methylation in A_m_ is not as well studied as m^6^A. One research group from Harvard University discovered Phosphorylated CTD Interacting Factor 1 (PCIF1) as the methyltransferase that generates m^6^A_m_ in mRNA. In this study they showed that, the methylation of adenosine is dependent on the presence of m^7^G in the adjacent position. This methyltransferase is incapable of methylating other adenosine outside the context of m^7^G presence in the 5'cap. The discovery of PCIF1 as the methyltransferase for m^6^A_m_ also facilitated the identification of alternative transcription start site (TSS) on mRNA since m^6^A_m_ is only located in this region 83, 84. Another research group almost at the same time using both *in vitro* and *in vivo* models discovered PCIF1 as the only methyl transferase for generating m^6^A_m_ 84.

#### m^5^C

m^5^C modification in E. coli rRNA is catalyzed by SAM-dependent methyltransferase enzyme, RsmB and RsmF 31. In higher eukaryotes DNA methyltransferaselike protein 2 (DNMT2) and members of the Nol1/Nop2/SUN (NSUN) family proteins play role as methyltransferases to catalyze the m^5^C modification. DNMT2 was identified as a specific methylation agent for cytosine at the 38 position on the tRNA anticodon loop 85. In yeast, the methyltransferase enzymes Trm4 (Ncl1), Nop2 and Rcm1 was reported to catalyze the m^5^C modification; whereas in humans, several other related proteins (e.g. p120, NSUN1/NOL1, NSUN2-7) are found to mediate the m^5^C modification 86. The NSUN family of proteins are assumed to be SAM-dependent methyl transferases that contain SAM binding site in their RNA-recognition motif 87. NSUN2 is the human orthologue of yeast Ncl1 protein which shows substrate specificity towards cytosine 34 in the wobble position of tRNA. It is localized mainly in the nucleoplasm and nucleus of the cell and specifically recognizes the intron-containing tRNAs 88. NSUN1 and NSUN5 are the human orthologues of yeast Nop2 and Rcm1which catalyze the methylation of cytoplasmic rRNA 28S subunit at cytosin4413 and 3761 respectively. NSUN3 and NSUN4 are synthesized in the ribosome and are transported to the mitochondria where they catalyze the methylation of the mitochondrial RNAs. NSUN4 installs m^5^C at the 911 position of the human rRNA 12S. NSUN3 on the other hand is responsible for methylation of C34 in the mitochondrial tRNA wobble position 87. NSUN6 is a tRNA methyltransferase and targets the cytosine C72 on the acceptor stem of human cytoplasmic tRNA 89. In mRNA, m^5^C modification was found to be catalyzed mainly by NSUN2 and is localized close to the translation initiation sites 90.

#### m^1^A

Another widely occurring RNA modification m^1^A is most commonly found in tRNA and conserved throughout the process of evolution. Since m^1^A modification is more abundant at position 58 of tRNA, m^1^A methyltransferase at this position is most extensively studied. The methyl group present on the m^1^A in tRNA is introduced by tRNA methyltransferase (MTase) using the S-adenosylmethionine (SAM) as the donor of the methyl group. Although the presence of the methyltransferase enzyme was first detected in 1962, the first purified MTase was isolated in 1976 from rat liver; the molecular weight of which has been determined as 95 kDa 91. The tRNA MTase contains two subunits named Trmt6 and Trmt61 and are encoded by the genes *TRMT6* and *TRMT61* 92. The gene products of *TRMT6* and *TRMT61* form a homoteramer complex, α2β2 of the two subunits where Trmt61 acts as the catalytic subunit to transfer the methyl group from SAM to A. Although Trmt61 acts as the catalyst for methyl transfer, both Trmt6 and Trmt61 subunits are essential for binding to RNA. In human, Trmt61A and Trmt6 subunits are orthologues of Trmt61 and Trmt6 subunits in *S. cerevisiae*. Human Trmt61 has two isoforms: Trmt61A and Trmt61B. Trmt61A is cytoplasmic tRNA specific whereas the Trmt61B is mitochondrial tRNA specific 39, 92. Another important m^1^A modification in human mitochondrial tRNA is at position 9 (m^1^A9). The enzyme for m^1^A9 modification is yet to be identified. Vilardo et al. reported the possibility of involvement of tRNA methyltransferase 10 C (TRMT10C) in m^1^A9 modification. TRMT10C is the human homolog of *S. cerevisiae* Trm10p. TRMT10C is a methyltransferase for m^1^G9 in human, however its role in m^1^A9 modification is yet to be confirmed 93.

#### A-to-I

The family of proteins that catalyzes the deamination of adenine in A-to-I editing is broadly named as adenosine deaminases acting on RNA or ADARs. ADARs catalyze the A-to-I conversion by hydrolytic removal of amide group from the 6-position of adenosine. In all organisms ADARs share common structural features: double stranded RNA binding motifs (dsRBMs), the number of which varies from species to species and a C-terminal catalytic domain which is highly conserved in all species 94. dsRBMs are required for RNA binding and substrate specificity whereas the catalytic domain is required for catalytic deamination. In mammals, three identical genes have been identified which encode for three subtypes of ADAR proteins- ADAR1, ADAR2 and ADAR3. The different ADAR proteins differ in their structure, substrate specificity and of course in their functional roles 95, 96. Besides the dsRBMs the amino-terminal of ADAR1contains Z-DNA- binding domains (ZBDs) that recognizes the left-hand DNA helix (in vertebrates) and also nuclear export/import signal domains (in mammals). In contrast, the N-terminal of ADAR2 is relatively shorter without any of these domains while the ADAR3 contains an arginine/lysine-rich motif (R domain) which is specific for binding to single stranded RNA (ssRNA) 96, 97. Although ADAR1 and ADAR2 have some differences in their target specificity towards adenosine in dsDNA, they share substrate specificity to some extent and are considered as the catalytically active members of ADAR family but the ADAR3 has been proven to be catalytically inactive. The gated ion-channel glutamate receptor (GluR) and serotonin receptor subtype 2C (5-HT_2C_R) are the most common targets for ADAR1 and ADAR2 where several specific editing sites are targeted by these two enzymes in these receptors 95, 97.

#### Ψ

The most abundant RNA modification psedouridne is introduced in RNA by the enzyme family named pseudouridine synthases or PUS enzymes. Six pseudouridine synthase families are known so far: TruA, TruB, TruD, RsuA, RIuA, and Pus10b. TruA, TruB, TruD, RsuA, RIuA are present in almost all organisms and extensively studied in bacteria whereas Pus10b is present only in humans and other eukaryotes. Although there is very little sequence similarity among them, all of the enzymes share the same structural features in their catalytic domains and hence a common mode of catalytic activity. Their substrate specificity is determined by differences in their N and C terminal domains 98. The PUS family enzymes are widely distributed in all domains of life and they have diverse subfamilies, some of which show specificity for specific sequence motifs. In humans, 13 different PUS enzymes have been reported that contribute to the pseudouridylation of which PUS1 shows specificity for mitochondrial tRNA at position 27 and 28 99. PUS enzymes can be categorized in two major classes: a) the RNA-dependent and b) RNA-independent PUSs. Although both type of enzymes act on tRNAs, mRNAs and snRNAs, RNA-independent PUSs mainly act on the prokaryotic rRNAs whereas the RNA-dependent PUSs target the eukaryotic rRNAs 100. Cbf5 in yeast, the human homolog of dyskerin (DKC1) is RNA dependent PUS which associates with Box H/ACA snoRNP where the snoRNA serves as a guide RNA and helps to recognize the RNA target for modification through base pairing. The RNA independent modifiers catalyze the modification in targets like tRNAs, rRNAs and other types of RNAs in a site specific manner through direct sequence recognition. PUS1 recognizes a specific sequence motif, HRU in human mRNA and dictates the mRNA modification. 59. Pus4 is the human orthologue of TruB1 which is found to play major role in Ψ modification in human mRNA along with another PUS enzyme, PUS7 101.

#### m^7^G

The common eukaryotic mRNA cap modification m^7^G is carried out by the heterodimeric enzyme complex METTL1-WDR4 in mammals which is the yeast homolog of Trm8 and Trm82 enzyme complex 73. The internal m^7^G modification in the tRNA and rRNA is carried out by the enzyme, WBSCR22 70.

### Erasers

#### m^6^A

In 2008 the first demethylating enzyme fat mass and obesity-associated (FTO) protein was discovered which brought new dimension in the RNA methylation study in mammals. This enzyme specifically removes methyl group from m^6^A by oxidative demethylation to revert it to adenosine in polyadenylated mRNAs and lncRNAs. As a member of the demethylating enzyme AlkB subfamily of Fe^2+^/α-ketoglutarate dependent dioxygenases, FTO catalyzes the oxidative demethylation of substrates from biological origin. The demethylating ALKB enzyme family recognizes the methylation on adenine and cytosine nucleotide bases in both single stranded DNA and RNA and to a smaller extent in the double stranded DNA which block DNA replication 13, 22, 23, 102. Initially it was presumed that FTO exerts its catalytic action on 3-methylthymidine (3-mT) and 3-methyluracil (3-mU) in ssDNA and ssRNA respectively; but recent studies showed that it can also convert m^6^A to two different intermediates: N6-hydroxymethyladenosine (hm^6^A) and N6-formyladenosine (f^6^A) by stepwise oxidative demethylation. This function of FTO is similar to that of ten eleven translocation (TET) enzyme which demethylates 5-methylcytosine (m^5^C) to 5-hydroxymethylcytosine (hm^5^C) and 5-formylcytosine (f^5^C) further to 5-carboxylcytosine (ca^5^C) in genomic DNA. This stepwise process is very important in active demethylation of DNA but the importance of similar demethylation in RNA is yet to be revealed. Like their methylating counter parts METTL3 and METTL14, FTO also co-localizes with them in the nuclear speckles which emphasizes the dynamicity and reversibility of the m^6^A modification process in mRNA 13. AlkB homologue5 (ALKBH5) was discovered as a second m^6^A demethylase in mammalian cells shortly after FTO. The human AlkB enzyme family has eight members which are designated as ALKBH1-ALKBH8. ALKBH5 and FTO share the similar substrate specificity (i.e. both target ssRNA) and demethylase activity on m^6^A, but unlike FTO, ALKBH5 demethylase directly converts m^6^A to adenosine without any intermediate. ALKBH5 also co-localizes in the nuclear speckles and controls the transport of mRNA between cytoplasm and nucleus, at the same time affecting the RNA metabolism 23.

#### Other erasers

m^6^A_m_ is demethylated by FTO in the same way as m^6^A and reverted to A_m_ . With *in vivo* and *in vitro* experiments Mauer et al. showed that m^6^A_m_ is a more preferred substrate for FTO than m^6^A. They also found that, m^6^A_m_ transcripts have more stability compared to mRNA transcripts with their non-methylated counterpart A_m_ or any other nucleotide. This enhanced stability is due to their resistance to mRNA decapping enzyme DCP2. This study suggests that m^6^A_m_ modification is a cap-associated dynamic and reversible mRNA modification 103.

Similar to m^6^A, certain ALKBH family proteins also remove methyl group from m^1^A. In one study it was shown that human AlkB homologue hABH3 can act as RNA demethylase. While another study showed ALKBH1can catalyze the demethylation of m^1^A. ALKBH1 demethylase can revert the methylation of m^1^A at 58 position on tRNA. The presence of a tRNA-binding motif has been identified on ALKBH1 which preferentially binds to the m^1^A on the tRNA stem loop structure 104. The methylation on m^1^A is also reported to be reversed by another member of DNA/RNA demethylase family ALKBH3 37. For m^5^C, A-to-I and Ψ; no eraser is identified yet.

### Readers

#### m^6^A

The ultimate outcome of the game of dynamic and reversible phenomenon 'methylation-demethylation' is exhibited by a third family of proteins called 'readers'. The family of proteins which specifically recognize m^6^A and bind to the methylated RNA to exhibit the functional outcomes are known as m^6^A readers. These proteins were identified for the first time using the antibodies targeting the methylated RNA and co-immunoprecipitation followed by mass spectrometry analysis 24. One of the well-known family of such proteins are YTH family proteins, which contain YTH-domain in their structure. Some of the members of this family designated as YTHDF1-YTHDF3 and YTHDC2 are localized mainly in the cytoplasmic region of the cell where they recognize the RNA transported out of the nuclear membrane after they are methylated by the RNA methyltransferases. Other proteins of YTH family (such as YTHDC1) were found to localize in the nucleus and recognize m^6^A in the nuclear region. The YTH domain contains a hydrophobic binding pocket for methyl group in the m^6^A at the recognition motif GG(m^6^A)C. These reader proteins have diverse profile of binding specificity for the cellular target mRNA which can be recognized by profiling their binding sites. YTHDF2 selectively binds to the target mRNA at G(m^6^A)C motif and mediates the mRNA decay. YTHDF1 and 3 coordinate each other in processing the m^6^A bearing mRNA for translation. However, the YTHDC2 was found to enhance the translational efficiency of m^6^A containing targets and decrease their abundance 105, 106. Heterogeneous ribonucleoproteins (hnRNPs) are also found to act as nuclear m^6^A readers in a recent study. One such protein hnRNPC was found to bind to an m^6^A altered stem-loop RNA structure which subsequently directs an alternative splicing of RNA. In absence of m^6^A the hnRNPC cannot bind with the RNA which affects its pattern of splicing 107. Another member of hnRNP protein family that lacks the YTH domain in its structure, hnRNPA2B1 is localized in nucleus and capable of binding m^6^A in mRNAs as well as miRNAs 108. Eukaryotic initiation factor 3 (eIF3) which directly binds to an m^6^A at 5' UTR of the mRNA is recognized as another reader protein playing vital role in protein translation under the stress condition 109. Recently, members of the insulin-like growth factor 2 mRNA-binding protein (IGF2BP) family was identified as a distinct m^6^A reader protein family. Three members of this family, IGF2BP 1-3 have been identified which can recognize the m^6^A consensus sequence in mRNA 110.

#### Readers of other modifications

The readers of m^1^A is similar to the readers of m^6^A. The m^6^A readers YTH domain containing family proteins YTHDF1-3 and YTHDC1-2 also act as m^1^A readers. But unlike the other members of the family, YTHDC2 cannot directly interact with the m^1^A 111. Aly/REF export factor (ALYREF) was thought to be the only protein to recognize and bind to m^5^C, therefore was the only known m^5^C reader protein which is localized mainly in the nucleus 90. But in a recent study another novel m^5^C reader, Y-box binding protein 1 (YBX1) has been discovered in an attempt to find a potential cytoplasmic m^5^C reader 112. However, no A-to-I or Ψ eraser has been identified as yet.

## RNA Modifications in Cancer

**Figure 5:**
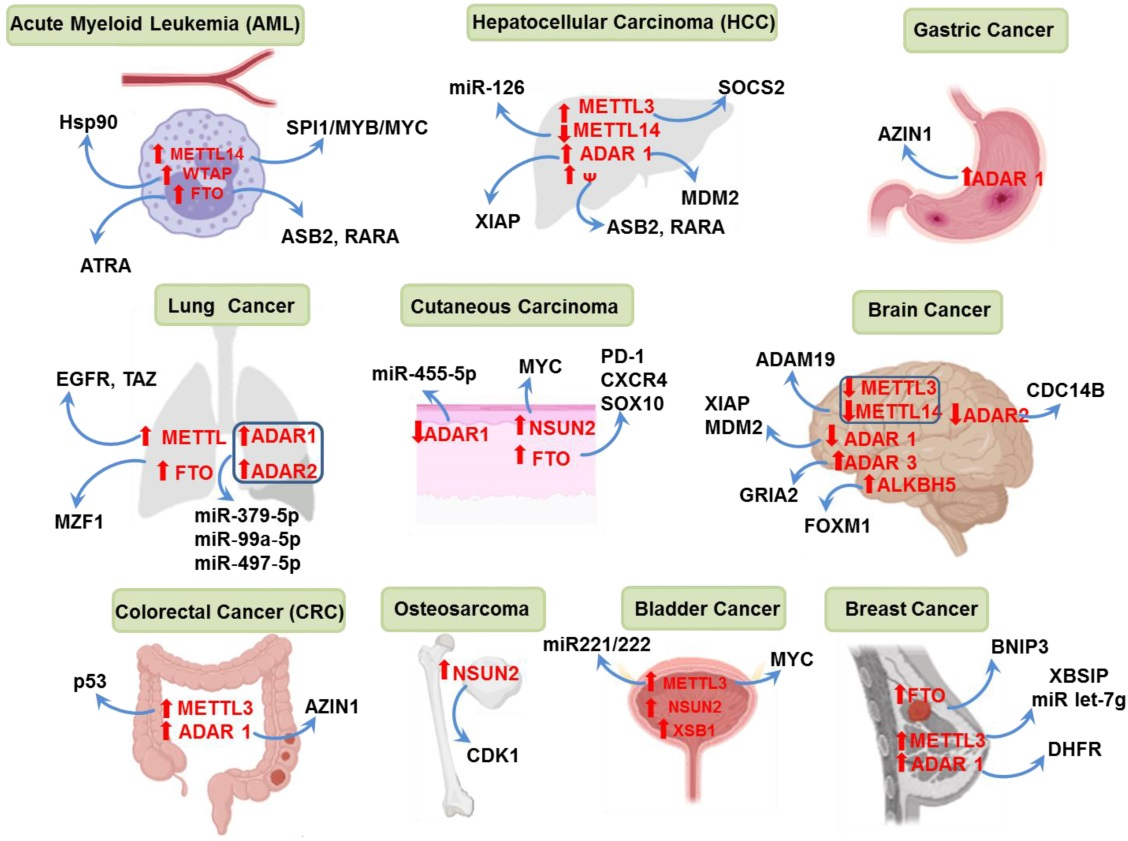
** RNA modifications in cancer: The RNA modification regulators and their targets in different cancer types.** Aberrant expression of RNA modification writers, readers and/or erasers is associated with different types of malignancies. The abnormally expressed regulatory proteins target a wide range of molecules causing their up/down regulation resulting in various types of cancer.

In the entire cellular transcriptome, RNA modification is versatile and observed in almost all types of cellular RNAs. In normal cellular processes, the extent of RNA modification is well regulated maintaining the homeostasis of RNA stability and decay. However, in cancer there is abnormal up/down regulation of modified RNA transcripts and their regulatory proteins that are correlated with the process of carcinogenesis (Table 2 and Figure 5).

### N6-methyladenosine (m^6^A) Modification in Cancer

In normal tissue development, the role of DNA methylation as a vital epigenetic factor is well established; however, association of m^6^A RNA modification as an epigenetic regulator in cancer is emerging now. RNA methylation at N6-methyladenosine (m^6^A) plays a central role in development of cancer which has been evidenced by several recent studies 77, 113. Alternative splice variants produced from pre-mRNA splicing are frequently observed in many cancer types, some of which are found to be mediated by m^6^A modification. In normal hematopoietic stem/progenitor cells (HSPCs), METTL14 is reported to be highly expressed while it is downregulated during the differentiation to myeloid cells. In acute myeloid leukemia (AML), METTL14 is also found to be highly expressed, silencing of which led to inhibition of cellular proliferation and reduced survival of AML cells. METTL14 knockdown also reduced the self-renewal capacity of leukemia stem cells. The oncogenic effect of METTL14 was due to an increase in the m^6^A level and activation of SPI1-METTL14-MYB/MYC signaling pathway 114. Another member of the RNA methylation complex, WTAP which acts as a cofactor of METTL3-METTL14 complex was reported to act as an oncoprotein in AML. Overexpression of WTAP was found in almost 32% of the AML cases where the patients are found to carry mutation in the NPM1 and FTL3-ITD genes. Depletion of WTAP showed a significant decrease in cell growth and promotion of cellular differentiation in AML 115, 116. Higher expression level of WTAP was also found in hepatocellular carcinoma (HCC) which is correlated with poor prognosis and low patient survival. Mechanistically, the protooncogene ETS1 was identified as the downstream target of WTAP. WTAP recruits Hu-Antigen R (HuR) to stabilize the ETS1 and suppress its oncogenic effect leading to HCC progression 117. The m^6^A writer METTL3 was reported to upregulate in hepatocellular carcinoma (HCC). Knockdown of METTL3 suppressed tumorigenicity and metastasis whereas overexpression promoted the HCC growth. The tumorigenic effect of METTL3 was due to METTL3-mediated m^6^A modification and suppression of SOCS2 mRNA expression. High expression of METTL3 is identified in patients with HCC which is correlated with tumorigenesis, vascular invasion and poor prognosis 118. METTL3 was also found to contribute to growth and drug resistance in colorectal cancer. The incorporation of m^6^A in p53 pre-mRNA transcript was observed in the p53 mutant colon cancer cells 119. Elevated level of METTL3 was found in clinical samples from breast cancer patients. Increase in m^6^A modification by METTL3 was shown to promote HBXIP expression which in turn rescued the miR let-7g-mediated METTL3 suppression in breast cancer cells. This mutual regulation of METTL3 and HBXIP promotes breast cancer progression 120. Upregulation of METTL3 is also observed in patients with ovarian cancer which is correlated with poor overall patient survival. Ovarian cancer cells with METTL3 overexpression exhibited increased cellular proliferation, invasiveness, metastasis and tumorigenicity in nude mice; which upon METTL3 silencing showed the opposite effects. METTL3 exerted the oncogenic effect in ovarian cancer cells by increasing the protein level of EMT-inducing AXL protein in cellular cytoplasm. In this case METTL3 mediated the increase of m^6^A methylation in AXL mRNA, thereby increasing translation of the protein 121. In bladder cancer (BCa) METTL3 overexpression has been correlated with increased cancer progression. METTL3 has been reported to promote the bladder cancer progression by AFF4/NF-κB/MYC signaling pathway where it upregulates the oncoprotein MYC expression in both direct and indirect manner. In one hand, METTL3 directly targets the MYC mRNA, thus stabilizing and upregulating MYC expression. On the other hand, METTL3 mediates m^6^A methylation and activation of NF-κB signaling molecules (IKBKB, RELA) and induces MYC transcriptional activity. METTL3 also modifies AFF4 transcript which binds to the promoter region of MYC mRNA and induces its transcription. This multiple transcriptional activation of MYC by METTL3 induces progression of BCa 122. In a different study, METTL3 was reported to interact with the microprocessor protein DGCR8 which accelerates the maturation of primary microRNA pri-miR221/222. Mature miR221/222 then binds to the PTEN and downregulates its expression thereby promoting BCa progression 123.

The demethylase FTO was found to enhance the cellular transformation and leukemogenesis and at the same time inhibit all-trans-retinoic acid (ATRA)-induced AML cell differentiation. The possible mechanism behind it is that, FTO caused demethylation of m^6^A in mRNA transcript and downregulation of Ankyrin repeat and SOCS box-containing 2 (ASB2) and retinoic acid receptor-α (RARA). During the normal process of hematopoiesis, ASB2 and RARA are upregulated and act as regulators of ATRA-induced cell differentiation. FTO inhibits the expression of ASB2 and RARA thus decreasing the differentiation of leukemic cells in AML 124. In a similar study FTO was found to overexpress in AML which significantly reduces the cellular m^6^A level and thus performs a vital oncogenic function by inhibiting ATRA-mediated AML cell differentiation 77. Interestingly, the expression of both m^6^A methyltransferase WTAP and demethylase FTO are increased in AML suggesting that, in different subtypes of AML m^6^A modification is tightly regulated by the mRNA methylation machinery 116. The m^6^A demethylase FTO was also found as a prognostic factor in lung squamous cell carcinoma (LUSC). Decreased m^6^A modification by FTO is associated with tumorigenesis of LUSC. FTO induces tumor progression by targeting MZF1, reducing the m^6^A in MZF1 transcript, increasing the stability and thus increased expression of MZF1 mRNA. Knockdown of FTO effectively reduced cell proliferation, invasiveness and increased apoptotic death 125. Increased expression of FTO and m^6^A demethylation of the protumorigenic mRNA transcripts (PD-1, CXCR4, and SOX10) accelerates the growth of melanoma cells and leads to reduced sensitivity against anti-PD-1 blockade immunotherapy. In absence of FTO, m^6^A in the target transcripts are recognized by YTHDF2 leading to their decay thereby sensitizing the cells towards interferon gamma (IFNγ) and anti-PD-1 treatment 126.

Decreased m^6^A methylation by FTO is also associated with breast cancer cell proliferation and metastasis. FTO-induced demethylation of the tumor suppressor BNIP3 mRNA in its 3'UTR causes degradation of the transcript thus eliminating the tumor suppressive effect of BNIP3 in breast cancer 127. The level of FTO was found downregulated in clinical samples of intrahepatic cholangiocarcinoma (ICC) and cells *in vitro*. An inverse correlation of FTO expression with the expression of CA19-9 and micro-vessel density (MVD) and stability of oncogenic TEAD2 mRNA was also reported 128.

In human lung cancer, METTL3 acts as a translation promoter for mRNAs of epidermal growth factor and Hippo pathway effector TAZ by recruiting the eukaryotic initiation factor 3 (eIF3). In addition to this, mRNA-ribonucleoprotein complex may replace the reader protein YTHDF1 for METTL3 at the m^6^A-containing site to promote EGFR and TAZ mRNA translation indicating that, increased m^6^A level may enhance the translation of proteins which promote lung cancer progression 77, 129. In one study it has been shown that, METTL14 knockdown in hepatocellular carcinoma (HCC) enhances the metastatic tumor progression by decreasing m^6^A mRNA level both *in vivo* and *in vitro*. Also, downregulation of METTL14 in HCC acts as a prognostic marker for recurrence of the disease. In this study, it was confirmed that METTL14 interacts with the microprocessor protein DGCR8 and has a positive modulatory effect on primary miR-126 in an m^6^A dependent manner. This study provides evidence of the involvement of m^6^A status in the cellular mRNA to the tumorigenesis in HCC through the regulation of microRNA signaling by METTL14 130. METTL3 and METTL14 knockdown was observed to upregulate the expression of several oncogenes such as ADAM19, EPHA3 and KLF4 in glioblastoma stem cells (GBCs). m^6^A immunoprecipitation followed by high-throughput sequencing confirmed the decrease in the overall level of m^6^A in ADAM19 mRNA 131. In a different study m^6^A demethylase ALKBH5 overexpression was observed in glioblastoma cells. Knockdown of ALKBH5 inhibited the proliferative capacity of GBCs. Silencing of ALKBH5 also altered the expression level of several ALKBH5 target genes which was evidenced from the integrated transcriptome and m^6^A immunoprecipitation followed by the sequence analysis of the mRNAs. Gene expression analysis in ALKBH5 knockdown cells have revealed forkhead box M1 (FOXM1) as the target gene for ALKBH5 which is the regulator of downstream genes controlled by ALKBH5. At the same time ALKBH5 knockdown increases m^6^A level in FOXM1 mRNA which lacks the regulation by HuR thus decreased FOXM1 expression 132.

### m^5^C Modification in Cancer

NSUN2 (Misu) is the most common m^5^C modifying enzyme in mammalian cells. Its association with tumorigenesis has been reported in several studies. In one study it has been shown that NSUN2 is over-expressed in tumors (benign papilloma and malignant squamous cell carcinoma) compared to normal cells. In this study NSUN2 was found as a downstream target for MYC which directly regulates cell proliferation 133. Knockdown of NSUN2 resulted in decreased m^5^C methylation and associated decrease in cell proliferation and migration of HEK293 cells. The genes associated with proliferation, migration and cell-cell adhesion were also affected by the NSUN2 knockdown 134. In another study NSUN2 was found to methylate the C1733 in the 3'UTR of the CDK1 mRNA thus elevating the CDK1 translational activity. CDK1 is an important cell cycle regulator, overexpression of which results in entry and progression of cell cycle 135. Association of m^5^C modification is reported in human urothelial carcinoma of bladder (UCB). Increased m^5^C methylation in several oncogenes is identified in UCB. The methylation is carried out by NSUN2 and is recognized by the m^5^C reader YBX1 which stabilizes the modified oncogenic mRNAs. Thus, NSUN2 and YBX1 in cooperation drive the tumorigenesis in UCB by hypermethylation of the mRNA targets 112. TRDMT1 (DNMT2), the tRNA m^5^C methyltransferase is associated with the regulation of various developmental processes through regulation of gene expression. Knockdown of TRDMT1 in human HEK293 cells resulted in inhibition of cellular proliferation and migration ability. The inhibitory effect of TRDMT1 knockdown was associated with the alteration of genes controlling cell cycle progression, RNA transport and degradation 136.

### A-to-I Modification in Cancer

Since A-to-I modification is more abundant in human brain than any other organs, the modification itself and the modification machinery are associated with various human neurological disorders including cancer. Grade III and IV astrocytomas, which are the most aggressive types of brain tumors, are associated with decreased level of editing activity by ADAR2 enzyme. The levels of ADAR2 was found significantly decreased in pediatric patients with aggressive astrocytomas, connecting it as a potential diagnostic marker in patients with higher grade astrocytoma or the recurrence of the disease 137, 138. ADAR2 and ADAR3 are both A-to-I editing enzymes in the brain. The balance between the modification enzymes is very important in maintaining the normal cellular homeostasis. RNA editing by ADAR2 in glutamate receptor ionotropic AMPA 2 (GRIA2) is essential for its normal calcium ion transportation. In glioblastoma, decreased level of ADAR2 and increased level of ADAR3 result in increased cell migration and invasiveness. In astrocytoma, increased ADAR3 inhibits the RNA editing in GRIA2 receptor by competitively binding and inhibiting ADAR2 139. Increased A-to-I editing in breast cancer was found to confer breast cancer cells resistance to methotrexate, an inhibitor of folate metabolism in cancer cells. Several A-to-I editing sites in 3'-UTR of dihydrofolate reductase (DHFR) mRNA was detected in such cells which is mediated by ADAR1 140. Overexpression of ADAR1 was also reported in oral squamous cell carcinoma (OSCC) and esophageal squamous cell carcinoma (ESCC) which is associated with the rapid tumor growth and poor disease prognosis in OSCC and ESCC patients 141, 142. Antizyme inhibitor 1 (AZIN1) is found to be one of the most frequently modified genes in colorectal cancer (CRC). Overexpression of ADAR1 and enhanced AZIN1 editing was found in CRC patients as well as gastric cancer and esophageal squamous cell carcinoma patients which serve as a prognostic factor in overall and disease-free survival in those patients 142-144. However, contradicting results of A-to-I modification are also reported in several studies where downregulation of the modifying enzyme ADAR1 is connected with the proapoptotic effects in cancer cells. Knockdown of ADAR1 decreased the A-to-I editing in the 3'-UTR of apoptosis inhibitors XIAP and MDM2 resulting in increased apoptotic cell death 145. Decreased A-to-I editing in certain microRNAs are attributed to the growth acceleration and invasiveness in melanoma 146, glioblastoma 147 and lung adenocarcinoma 148.

### Other RNA Modifications in Cancer

The specific m^1^A demethylase ALKBH3 which is originally known as prostate cancer antigen-1 (PCA-1) is present in high abundance in prostate cancer. Knockdown of ALKBH3 was found to decrease the proliferation of prostate cancer cell proliferation and tumor growth *in vivo*. ALKBH3 has also been found to be highly expressed in other cancer types such as pancreatic cancer, renal cell carcinoma and non-small-cell lung carcinoma etc.^149^.

Mutation in the snoRNA-dependent pseudouridine synthase DKC1 is associated with a heriditary bone marrow failure syndrome Dyskeratosis Congenita (DC) which leads to various cutaneous and noncutaneous abnormalities including premature ageing and cancer 150. Depletion of snoRNA is also associated with RAS mediated oncongenic transformation. Loss of an H/ACA snoRNA, SNORA which directs site specific Ψ modification in 18s rRNA subunit results in RAS-induced development of liver cancer in mice. Low expression of the same snoRNA in human hepatocellular carcinoma showed increased lipid accumulation and was associated with poor patient survival 151. An opposite effect of H/ACA snoRNAs was observed in prostate cancer cells. Elevated level of Dyskerin (DKC1) and H/ACA snoRNAs and therefore high level of Ψ was found in the *in vitro* studies with prostate cancer cells 152. In a recent study, gene expression profiling for prognosis of glioma revealed an increased expression of psudouridylation writer PUS7 among several other genes 153.

METTL1, writer of the common 5'-cap modification m^7^G in mRNA and internal modification in other non-coding RNAs, is found upregulated in hepatocellular carcinoma (HCC). Overexpression of METTL1 is correlated with poor overall survival in HCC patients. METTL1 overexpression also showed increased cell proliferation and migration in *in vitro* studies. The oncogenic effect of METTL1 is exhibited via the PTEN/AKT signaling pathway. METTL1 downregulation activates the PTEN signaling with subsequent attenuation of AKT activity. Combined downregulation of METTL1 and upregulation of PTEN improved the overall patient survival. Therefore, METTL1 serves as a potential prognostic marker for HCC 154. The writers of m^5^C and m^7^G, NSUN2 and METTL1 are both tRNA modifiers which are found concurrently overexpressed in human cancers. Combined knockdown of both NSUN2 and METTL1 in HeLa cells increased their sensitivity to chemotherapeutic drug 5-fluorouracil (5-FU) 155.

## RNA Modification in Cancer Stem Cell Generation

Cells with special capabilities of self-renewal and the ability to differentiate into diverse progenies in response to specialized signals are termed stem cells. Different RNA modifications and their regulators have been reported to be associated with pluripotent attribuites of the normal stem/progenitor cells 156, 157. The m^6^A Writer protein METTL3 plays a vital role in regulating pluripotency of the normal stem cells. Depletion of METTL3 in naïve embryonic stem cells (ESCs) resulted in defective differentiation of embryonic bodies and neuronal stem cell maturation. Thus, lack of METTL3 maintains the pluripotency in naïve ESCs. The opposite effect of METT3 depletion was observed in primed pluripotent stem cells. In this case METT3 depletion diminished self-renewal capability and accelerated the stem cell differentiation 158. m^6^A methylation catalyzed by METTL3 and METTL14 has been found to be involved in embryonic stem cell formation in mouse 23. m^6^A RNA modification and its regulators also play crucial roles in maintaining pluripotency in porcine induced pluripotent stem cells (piPSC). piPSCs maintain pluripotent attributes through METTL3 regulated activation of JAK2-STAT3 signaling pathway. METTL3 mediates the m^6^A modification in both JAK2 and the negative regulator of the JAK2-STAT3 pathway, SOCS3 which causes decreased SOCS3 expression resulting in rescue of the repressive effect of SOCS3 on this pathway. In this case, m^6^A modified JAK2 mRNA is recognized and stabilized by YTHDF1, whereas the SOCS3 mRNA is degraded by YTHDF2 159. The same signaling pathway was reported to be associated with adipogenesis in a FTO dependent manner where it is shown that FTO demethylates the JAK2 mRNA which activates the STAT3 for adipocyte differentiation by C/EBPβ. In absence of FTO, the m^6^A modified JAK2 mRNA is recognized and degraded by YTHDF2 160. In contrary to the previous findings, METTL3 inhibited differentiation of bone marrow stem cells (BMSCs) to adipocytes was found to be associated with inactivation of JAK1/STAT5/C/EBPβ pathway. METTL3 incorporates m^6^A modification to JAK1 mRNA which is recognized by YTHDF2, leading to JAK1 degradation and inhibition of STAT5 phosphorylation. Failure of STAT5 phosphorylation inhibits STAT5 mediated activation of C/EBPβ mRNA transcription resulting in failure to adipocyte differentiation to adipocytes 161. The Ψ writer, DKC1 is reported to play role in transcriptional coactivation of pluripotency factors OCT4 and SOX2 in embryonic stem cells 151.

In addition to their roles in maintaining pluripotency in the normal stem cells, RNA modifications and their regulatory proteins are also found to contribute to the stemness attributes of the cancer cells (Table 3). In cancer cell population there are certain subpopulations of cells that are capable of self-renewal are known as cancer stem cells (CSCs). Due to their pluripotent character, CSCs impose challenges to the chemotherapy by developing resistance and thereby recurrence of cancer after eradication 162.

m^6^A RNA modification and its regulators play crucial role in CSC generation and maintenance. METTL14 has been found highly expressed in normal hematopoietic stem/progenitor cells (HSPCs) which is downregulated in the process of normal myelopoiesis. In acute myeloid leukemia (AML), the m^6^A writer METTL14 was found upregulated. Ablation of METTL14 reduced the immortalization of the cells *in vitro* and decreased the tumorigenicity of the AML cells *in vivo*. The tumorigenicity and stemness of AML cells were due to METTL14-mediated methylation and increased stability of the MYB and MYC mRNA transcripts. Knockdown of MYB and MYC resulted in myeloid differentiation whereas ectopic expression of these two genes restored the self-renewal properties of the leukemic stem cells (LSCs) in AML 114.The m^6^A reader protein YTHDF2 is also reported to increase the leukemic stem cells (LSCs) in AML. This reader protein was found to decrease the half-life of the target transcripts (e.g TNF receptor 2 (TNFR2)) thus eliminating their repressive effect on LSCs 163. Another m^6^A writer METTL3 was also reported to be overexpressed in AML leading to accelerated leukemia progression. Increased m^6^A methylation of c-MYC, BCL2 and PTEN mRNAs by METTL3 increased translation of these proteins and downregulated the phosphor-Akt contributing to the stemness behavior of AML cells 164. In glioblastoma, overexpression of METTL3 was reported to be crucial in maintaining the stemness and dedifferentiation capability of glioblastoma stem cells (GSCs). In this study, three specific locations in the 3'-UTR of SOX2 mRNA was found to be the target for m^6^A modification. Silencing METTL3 sensitized the GSCs to γ-irradiation and reduced the neurosphere formation 165. The opposite effect of m^6^A methylation in glioblastoma was observed in other studies. The increased expression of METTL3 and METTL14 or decreased demethylase FTO resulting in increased m^6^A methylation suppressed the growth and self-renewal potential of GSCs. This is evidenced by knockdown of METTL3 and METTL14 and subsequent increase in several oncogenes (ADAM19, EPHA3, and KLF4) and a decrease in tumor suppressor genes (CDKN2A, BRCA2, and TP53I11) 131. Another similar study reported that m^6^A demethylase enzyme ALKBH5 is overexpressed in GSCs. ALKBH5 mediated the cancer progression 3 and stemness behavior of GSCs through demethylation of the transcription factor FOXM1 nascent transcripts, thus increasing FOXM1 expression 132. Similar result was also observed in breast cancer cells where ALKBH5-mediated m^6^A demethylation of NANOG mRNA enhanced the breast cancer stem cell (BCSC) in hypoxic condition. ALKBH5 upregulation showed decreased m^6^A level in pluripotency induction marker NANOG mRNA in breast cancer stem cells (BCSCs). In this case, ALKBH5 expression is induced by hypoxia which reduces the adenosine methylation in NANOG mRNA transcript and increases its stability and expression of protein level, ultimately increasing the stemness behavior in BCSCs. Inhibition of ALKBH5 expression can be a potential therapeutic strategy to downregulate the NANOG expression and block the recurrence of breast cancer by BCSCs 166.

ADAR1 is the modification enzyme for the A to I modification and ADAR1 overexpression in oral squamous cell carcinoma (OSCC) contributes to the promotion and maintenance of cancer stem cells. In OSCC cells ADAR1 was found to physically interact with the miRNAs processing machinery Dicer and six miRNAs to promote oncogenesis and stemness 141. Elevated expression of ADAR1 level and AZIN1 RNA modification is observed in colorectal cancer tissues from patients compared to normal colon epithelial cells. Enhanced A-to-I editing of AZIN1 mediated by ADAR1 is associated with stemness attribute and metastatic potential of colorectal adenocarcinoma as evidenced by increased OCT4 and SOX2 levels in these cells 143.

The m^5^C and pseudouridine modifications in mRNA are also reported to be associated with pluripotency of the mammalian cells. With the transfection of m^5^C- and pseudouridine-modified synthetic mRNAs encoding the four pluripotency factors (Yamanaka factors) KLF4, c-MYC, OCT4, and SOX2, Warren and colleagues were able to reprogram the differentiated human cells to pluripotent cells 167. The m^5^C modification was identified in higher abundance in the undifferentiated pluripotent embryonic stem cell (ESC) mRNAs which are associated with cell cycle regulation, RNA and chromatin modification etc. 168. The m^5^C writer NSUN2 is a downstream target for c-Myc which is a well-known regulator of stem cell differentiation. It is found to be a determinant for the self-renewal and differentiation of epidermal hair follicular stem cells. In epidermal cells the NSUN2 expression is maintained in a low level; however when the cells are committed to differentiation, the level is increased 169. In squamous cell carcinoma, inhibition of m^5^C modification by down regulation of NSUN2 promotes cancer stem cell generation and tumorigenesis 170.

RNA pseudouridylation has a profound effect on cellular protein synthesis and determination of cell fate. The Pseudouridine writer, PUS7 is found to be a critical regulator of protein translation controlling stem cell growth and differentiation. PUS7 dysfunction is associated with perturbed protein synthesis in embryonic stem cell (ESC) resulting in increased hematological disorders. Impaired PUS7 function retards ESC differentiation and promotes malignant transformation to generate aggressive acute myeloid leukemia (AML) stem cells 171.

## Functional RNA Modification Machinery as Anticancer Drug Targets

The knowledge of base modifications in RNA molecule is relatively old but their role in vital cellular processes and disease conditions has only been revealed in recent years. Especially the implications of RNA modifications and their regulatory machinery in cancers are still largely unknown. This has triggered a tremendous surge of research efforts in last couple of years to reveal the involvement of RNA posttranscriptional modifications in cancer and a handful of literatures have been published on this issue. Based on the published reports, the modified RNA bases and their regulators have been found to be up/down regulated in a number of cancer cases which contribute to overall cancer progression and poor patient survival (Table 2). This has raised the question, can these oncogenic players be potential targets for developing new anticancer therapeutics? Most of the efforts to this end is focused mainly on the RNA modifying proteins (writers, erasers and readers) due to the advantage of the availability of high resolution crystal structures of these proteins. The crystal structures of these proteins facilitate the structure-based drug design where ligands are designed for potential binding sites using the powerful computational tools. Like other protein targets, the structure and ligand based drug design are now being used to design molecules that may interfere with the binding interaction between these proteins and their target molecules 172, 173.

The approaches for targeting the RNA modification machinery as anticancer drug targets are still in its infancy and most of the effort is focused mainly on the proteins involved in RNA methylations (methyltransferases, demethylators and other RNA binding proteins). The RNA methyltransferases are mostly S-adenosyl-L-methionine (SAM) dependent methyl transferases which catalyze the transfer of the methyl group using SAM as the substrate. There are at least five distinct classes of SAM-dependent methyltransferases (Class I to V), but the majority of the enzymes belong to class I and IV. The methyltransferases responsible for m^6^A, m^1^A, m^5^C, m^7^G and other methylations fall under Class I category which is the largest among all other groups 174, 175. Although selective DNA methyltranferase inhibitors have been developed (Azacytidine and Decitabine) and are approved for treatment of hematological malignancies, no specific inhibitors for RNA methyltransferases have been identified yet. However, because of the structural similarity among the SAM dependent methyltransferases, there is a high potential for the development of specific RNA methyltransferase inhibitors 176. In an effort to find potential small molecular ligand for m^6^A-methyltransferase complex METTL3/METTL14/WTAP, Selberg et al. used *in silico*-based drug discovery method and designed molecules that can specifically bind the complex. They further tested the molecules for their binding affinity and kinetics with the complex as well as their effects on the enzyme activity 172. Bioactive phytochemicals may also be a potential source of anticancer drugs targeting RNA posttranscriptional modifications. Sulforaphane (SFN), an isothiocyanate which is abundant in the cruciferous vegetables, have been shown to induce cell cycle arrest, oxidative stress and genotoxicity to different phenotypic varieties of breast cancer cells by decreasing global DNA and m^6^A RNA methylation 177.

Most of the known RNA demethylases belong to the Fe^2+^/α-ketoglutarate dioxygenase family of enzymes (FTO and ALKBH family). All the members of this family share a common jellyroll β‑sheet coordinating Fe^2+^ binding which is critical for their optimal functionality. Apart from this, individual members of the family have additional domains 178. These demethylases require the Fe^2+^ as a co-factor and 2-oxoglutarate (2OG) as a co-substrate to carry out their demethylation functions 179. Designs of inhibitors for these proteins are based mainly on targeting the binding sites of Fe^2+^ and 2OG. With biochemical and crystallographic analysis several small molecules targeting the 2OG binding site has been identified which upon optimization can give rise to molecules with higher potency and substrate selectivity 173. 1-(5-methyl-1H-benzimidazol-2-yl)-4-benzyl-3-methyl-1H-pyrazol-5-ol (HUHS015), an Inhibitor for the m^1^A demethylase ALKBH3 (also known as prostate cancer specific antigen (PCA-1)) is derive from screening of a number of 1-aryl-3,4-substituted-1H-pyrazol-5-ol derivatives. This specific inhibitor showed promising anticancer activity against prostate cancer cells in culture and mouse tumor xenografts 180. Rhein, a plant based natural compound present in rhubarb (*Rheum palmatum*) was found as an FTO inhibitor which occupies the Fe^2+^ and 2OG along with the nucleotide binding sites 181.

The known reader proteins are the RNA binding proteins which belong to YTH domain containing family of proteins. The YTH domain is well conserved in all three domains of life; the unique features of which may be exploited in inhibitor design in the approach of new drug discovery 182, 183. No inhibitor or antagonist for the RNA reader proteins have been identified to date.

## Concluding Remarks and Future Perspectives

RNA modifications have been established as key players of diverse cellular processes which are determined by the factors like - subcellular localization of the modifying enzyme, the enzyme that recognizes the modification etc. Depending on the modifying enzyme and its subcellular localization as well as the modification reader, the modification causes changes in the translational efficiency of the transcripts, regulates their shuttling between cytoplasm and nucleus, determines their stability or recruits splicing factors thereby determining splice variants 184-188. Since the RNA modifying enzymes can incorporate modifications in both coding and non-coding RNA moieties, many of these modifications contribute to additional binding capabilities with diverse cellular components expanding their arrays of regulation.

In addition to their roles in regulating normal cellular processes, RNA modifications are also found to play roles in carcinogenesis and confer stemness to the cancerous cell sub populations. On one hand, RNA modifications can contribute to cancer progression by decreasing the stability of the suppressors of the oncogenes thereby eliminating their inhibitory effects, on the other hand they can enhance the stability and expression of the protooncogenic transcripts 118, 132. How cancer cells selectively modify the target mRNA transcripts for driving malignant transformation is a matter to ponder upon. In many cases the oncogenic effects of the RNA modifications are due to incorporation of the modified nucleotides in the non-coding RNAs such as miRs and lncRNAs affecting their binding interactions with the target mRNA transcripts. These modifications may increase or decrease their binding affinity with specific sequence motifs on the target mRNAs which may determine their stability or contribute to their transcriptional activation/ repression contributing to tumorigenesis and/or stemness 130, 148. The pluripotent attributes of CSCs may also be associated with a particular type of RNA modification or its regulator(s). Modified RNA signatures and their regulatory proteins may serve as potential biomarkers for particular types of cancers or CSCs that can be targeted for combating therapeutically refractory tumors. 130, 153, 180. However, to predict a particular modification or regulator as a biomarker in a specific cancer cell type or subpopulation requires additional insight- whether it is unique in cancerous cells and not in the surrounding normal cells. Also, it requires reliable detection techniques to identify the modification with adequate sensitivity to avoid false positive results and distinguish artifacts from actual positive results. RNA modifiers incorporate modifications by recognizing specific sequence motifs in the target transcripts; however the target nucleotide is not always modified in that particular sequence. Which factor determines the location for the modification to occur is a question to be answered. Another big challenge in this field is that, the occurrence and contributions of many of the modifications in cancers although identified, their molecular targets in pathogenesis of cancers are yet to be revealed. Moreover, the enormous amount of sequencing data generated while studying the modifications also needs to be carefully analyzed and validated to confirm the findings. In addition, environmental pollutants are important cancer etiological factors promoting cancer initiation and progression. However, whether and how environmental exposure triggers abnormal RNA modifications and their roles in environmental carcinogenesis await further investigations.

## Figures and Tables

**Table 1:** Regulators of RNA modifications.

Modification	Writer	Eraser	Reader
**m^6^A**	Methyltransferase-like 3 (METTL3)	Fat mass and obesity-associated protein (FTO)	YTH domain family protein 1-3 (YTHDF1-3)
	Methyltransferase-like 14 (METTL14)	AlkB homologue5 (ALKBH5)	YTH domain-containing protein 1-2 (YTHDC1-2)
	Methyltransferase-like 16 (METTL16)		Eukaryotic initiation factor 3 (eIF3)
	Wilms' tumour 1-associating protein (WTAP)		Insulin-like growth factor 2 mRNA-binding protein 1-3 (IGF2BP1-3)
	RNA binding motifs protein 15 (RBM15)		Heterogenous ribonucleoprotein C (hnRNPC)
	RNA binding motifs protein 15B (RBM15B)		Heterogenous ribonucleoprotein A2B1 (hnRNPA2B1)
	Vir-like m^6^A methyltransferase associated (VIRMA)/KIAA1429		
	HAKAI		
	Zinc finger CCCH domain-containing protein 13 (ZC3H13)		
	Methyltransferase-like 4 (METTL4)^?^		
**m^6^A_m_**	Phosphorylated CTD Interacting Factor 1 (PCIF1)	Fat mass and obesity-associated protein (FTO)	Unknown
**m^1^A**	tRNA MTase6 (Trmt6)	Human AlkB homologue3 (hABH3)	YTH domain family protein 1-3 (YTHDF1-3)
	tRNA MTase61A (Trmt61A)	AlkB homologue1 (ALKBH1)	YTH domain-containing protein 1-2 (YTHDC1-2)
	tRNA MTase10C (Trmt10C)	AlkB homologue3 (ALKBH3)	
**A-to-I**	Adenosine deaminases acting on RNA1-3 (ADAR1-3)	Unknown	Unknown
**m^5^C**	DNA methyltransferaselike protein 2 (DNMT2)	Unknown	Aly/REF export factor (ALYREF)
	Nol1/Nop2/SUN (NSUN) family protein 1-7 (NSUN1-7)		Y-box binding protein 1 (YBX1)
**Psedouridne (Ψ)**	Pseudouridine synthase 1-4 (PUS1-PUS4), PUS6, PUS7, PUS9, PUS10	Unknown	Unknown
	RPUSD1-4		
	Dyskerin (DKC1)		
**m^7^G**	Methyltransferase-like 1 (METTL1)	Unknown	Unknown
	WBSCR22		

**Table 2:** RNA modification in Cancer

Modification	Type of Cancer	Mediator	Effect	Mechanism	Reference
**m^6^A**	Acute myeloid leukemia (AML)	METTL14	Overexpression	Activation of SPI1-METTL14-MYB/MYC signaling pathway	114
	Acute myeloid leukemia (AML)	WTAP	Overexpression	Hsp90 mediated WTAP stabilization	115
	Hepatocellular carcinoma (HCC)	WTAP	Overexpression	Hu-Antigen R (HuR) mediated stabilization and suppression of protooncogene ETS1	117
	Hepatocellular carcinoma (HCC)	METTL3	Overexpression	m^6^A modification and suppression of SOCS2 mRNA expression	118
	Breast cancer	METTL3	Overexpression	HBXIP overexpression and miR let-7g downregulation	120
	Lung cancer	METTL3	Overexpression	Upregulation of EGFR and Hippo pathway effector TAZ	129
	Colorectal cancer	METTL3	Overexpression	m^6^A methylation in the p53 pre-mRNA transcript	119
	Ovarian Cancer	METTL3	Overexpression	Increased m^6^A methylation and translation of AXL mRNA	121
	Bladder cancer (BCa)	METTL3	Overexpression	Activation of AFF4/NF-κB/MYC signaling pathway	122
	Bladder cancer (BCa)	METTL3	Overexpression	Maturation of microRNA miR221/222	123
	Acute myeloid leukemia (AML)	FTO	Overexpression	Ankyrin repeat and SOCS box-containing 2 (ASB2) and Retinoic acid receptor-α (RARA) mRNA demethylation and downregulation	124
	Acute myeloid leukemia (AML)	FTO	Overexpression	ATRA-mediated AML cell differentiation	77
	Lung squamous cell carcinoma (LUSC)	FTO	Overexpression	Decreased m^6^A methylation and MZF1 mRNA stabilization	125
	Melanoma	FTO	Overexpression	Decreased m^6^A methylation of protumorigenic PD-1, CXCR4, and SOX10 mRNA	126
	Breast cancer	FTO	Overexpression	m^6^A methylation of tumor suppressor BNIP3 mRNA	127
	Intrahepatic cholangiocarcinoma (ICC)	FTO	Downregulation	Decreased TEAD2 mRNA stability and CA19-9 and micro-vessel density (MVD) expression	128
	Hepatocellular carcinoma (HCC)	METTL14	Knockdown	m^6^A dependent miR-126 modulation	130
	Glioblastoma	METTL3 METTL14	Knockdown	Decrease m^6^A methylation of ADAM19 mRNA	131
	Glioblastoma	ALKBH5	Overexpression	Forkhead box M1 (FOXM1) downregulation	132
**m^1^A**	Prostate cancer Pancreatic cancer Renal cell carcinoma Non-small-cell lung carcinoma	ALKBH3	Overexpression	Increased expression of target proteins	149
**A-to-I**	Astrocytoma	ADAR2	Downregulation	Modulation of the CDC14B/Skp2/p21/p27 axis	138
	Glioblastoma	ADAR3	Overexpression	A-to-I editing in glutamate receptor ionotropic AMPA 2 (GRIA2)	139
	Breast cancer	ADAR1	Overexpression	A-to-I editing in 3'-UTR of dihydrofolate reductase (DHFR) mRNA	140
	Esophageal squamous cell carcinoma (ESCC)	ADAR1	Overexpression	A-to-I hyperediting of FLNB and AZIN1genes	142
	Oral squamous cell carcinoma (OSCC)	ADAR1	Overexpression	Increased expression of oncogenic miRs	141
	Colorectal cancer (CRC)	ADAR1	Overexpression	Elevated AZIN1 RNA editing	143
	Gastric cancer	ADAR1	Overexpression	A-to-I hyperediting of AZIN1 mRNA	144
	Melanoma	ADAR1	Downregulation	Reduced A-to-I editing of miR-455-5p	146
	Glioblastoma	ADAR1	Downregulation	Reduced A-to-I editing of miR-376a	147
	Lung adenocarcinoma	ADAR1 ADAR2	Downregulation	Reduced A-to-I editing of miR‐379‐5p, miR‐99a‐5p, and miR‐497‐5p	148
	Glioblastoma Hepatocellular carcinoma (HCC)	ADAR1	Downregulation	Decreased A-to-I editing in 3'-UTR of XIAP and MDM2	145
**m^5^C**	Benign papilloma Malignant squamous cell carcinoma	NSUN2	Overexpression	MYC-induced cellular proliferation	133
	Osteosarcoma	NSUN2	Overexpression	m^5^C methylation in 3'UTR of CDK1 mRNA and elevating CDK1 translational activity	135
	Urothelial carcinoma of bladder (UCB)	NSUN2 YBX1	Overexpression	m^5^C hypermethylation of the mRNA targets	112
**Psedouridne (Ψ)**	Prostate cancer	DKC1	Overexpression	Increased H/ACA snoRNAs	152
	Hepatocellular carcinoma (HCC)		Decreased Ψ	Loss of snoRNA, SNORA	151
**m^7^G**	Hepatocellular carcinoma (HCC)	METTL1	Overexpression	PTEN downregulation and activation of AKT signaling pathway.	

**Table 3:** RNA modification in cancer stem cell development.

CSC	Change	RNA Modification	Modifying Component	Target	Functional Consequences	Reference
Leukemic stem cells (LSCs)	Increase	m^6^A methylation	METTL14	MYB and MYC mRNA	↑ mRNA stability	114
Leukemic stem cells (LSCs)	Increase	m^6^A methylation	METTL3	c-MYC, BCL2, PTEN and p-Akt	↑ c-MYC, BCL2, PTEN ↓ p-Akt	164
Leukemic stem cells (LSCs)	Increase	m^6^A methylation recognition	YTHDF2	TNF receptor 2 (TNFR2)	↓ TNFR2 half-life and related transcripts	163
Glioblastoma stem cells (GSCs)	Increase	m^6^A methylation	METTL3	SOX2	SOX2 mRNA stabilization	165
Glioblastoma stem cells (GSCs)	Decrease	m^6^A methylation	↑ METTL3, METTL14 ↓ FTO	Several oncogenes and tumor suppressors	↑ Oncogrenes (ADAM19, EPHA3, and KLF4) ↓ Tumor suppressors (CDKN2A, BRCA2, and TP53I11)	131
Glioblastoma stem cells (GSCs)	Increase	m^6^A demethylation	ALKBH5	FOXM1 nascent transcript	↑ FOXM1 expression	132
Breast cancer stem cell (BCSC)	Increase	m^6^A demethylation	ALKBH5	NANOG mRNA	↑ NANOG stability and expression	166
Oral squamous cell carcinoma-cancer stem cells (OSCC-CSCs)	Increase	A-to-I editing	ADAR1	Dicer miRNAs processing machinery	↑ Oncogenic miRs	141
Colorectal Cancer Stem Cells (CRC-CSCs)	Increase	A-to-I editing	ADAR1	AZIN1 RNA	↑ AZIN1 RNA editing	143
Cutaneous squamous cell carcinoma stem cells	Increase	m^5^C methylation	NSUN2	tRNAs	Inhibits tRNA cleavage	170
Leukemic stem cells (LSCs)	Increase	Pseudouridylation	PUS7	tRNA-derived small fragments (tRFs)	↓ Hematopoietic stem cell differentiation	171
